# X-chromosome inactivation in human iPSCs provides insight into X-regulated gene expression in autosomes

**DOI:** 10.1186/s13059-024-03286-8

**Published:** 2024-05-31

**Authors:** Hande Topa, Clara Benoit-Pilven, Taru Tukiainen, Olli Pietiläinen

**Affiliations:** 1 Neuroscience Center, Helsinki Institute of Life Science, University of Helsinki, Helsinki, Finland; 2 Institute for Molecular Medicine Finland, Helsinki Institute of Life Science, University of Helsinki, Helsinki, Finland; 3 The Stanley Center for Psychiatric Research at the Broad Institute, of MIT and Harvard, Cambridge, MA USA

**Keywords:** X chromosome inactivation, hiPSC, Sex differences, RNA-seq, Allele-specific expression, *XIST*-bound autosomal genes, X chromosome-regulated gene expression

## Abstract

**Background:**

Variation in X chromosome inactivation (XCI) in human-induced pluripotent stem cells (hiPSCs) can impact their ability to model biological sex biases. The gene-wise landscape of X chromosome gene dosage remains unresolved in female hiPSCs. To characterize patterns of de-repression and escape from inactivation, we performed a systematic survey of allele specific expression in 165 female hiPSC lines.

**Results:**

XCI erosion is non-random and primarily affects genes that escape XCI in human tissues. Individual genes and cell lines vary in the frequency and degree of de-repression. Bi-allelic expression increases gradually after modest decrease of *XIST* in cultures, whose loss is commonly used to mark lines with eroded XCI. We identify three clusters of female lines at different stages of XCI. Increased XCI erosion amplifies female-biased expression at hypomethylated sites and regions normally occupied by repressive histone marks, lowering male-biased differences in the X chromosome. In autosomes, erosion modifies sex differences in a dose-dependent way. Male-biased genes are enriched for hypermethylated regions, and de-repression of XIST-bound autosomal genes in female lines attenuates normal male-biased gene expression in eroded lines. XCI erosion can compensate for a dominant loss of function effect in several disease genes.

**Conclusions:**

We present a comprehensive view of X chromosome gene dosage in hiPSCs and implicate a direct mechanism for XCI erosion in regulating autosomal gene expression *in trans*. The uncommon and variable reactivation of X chromosome genes in female hiPSCs can provide insight into X chromosome’s role in regulating gene expression and sex differences in humans.

**Supplementary Information:**

The online version contains supplementary material available at 10.1186/s13059-024-03286-8.

## Background

There is great interest for using human-induced pluripotent stem cells (hiPSCs) to understand cellular functions underlying male–female diversity in clinically relevant traits and disorders showing sex biases. Different female lines can vary in their epigenetic landscape in the X chromosome, which can influence their utility especially for modeling cellular functions that lead to X-linked disorders [1–5]. However, little is known about how X chromosome inactivation (XCI), a dosage compensation mechanism of gene expression, affects individual genes in female hiPSCs. XCI silences transcription from the extra copy of chromosome X in females (XX) to balance the expression dosage with that of XY males [6]. XCI in humans takes place during early embryonic development [7, 8]. From there on, it is forwarded to all descending cells. XCI is random in humans, so that typically approximately half of the embryonic cells will pass on a transcriptionally active paternal copy and the rest will have an active maternal X chromosome [9]. Intriguingly, XCI does not equally cover the whole chromosome but more than 20% of genes are reported to be transcribed to varying degrees also from the inactive copy of the X chromosome [10]. Escape from XCI can subsequently result in sex-biased gene expression and may contribute to phenotypic diversity in males and females [10, 11].

During reprogramming, hiPSCs undergo global remodeling of their epigenetic state, but they retain the same inactive and active copy of chromosome X as their somatic progenitor (Xi and Xa, respectively) [2, 12]. Instead of being mosaic for Xi, however, hiPSC cultures are reported to be clonal, suggesting that each line descends from a single somatic progenitor cell [1, 2, 12]. Nevertheless, hiPSCs display epigenetic variation including erosion of XCI in prolonged cultures [5]. The erosion is associated with the loss of expression of the long non-coding RNA *XIST*, which is required to achieve XCI [2, 3, 13]. Previously, low levels of *XIST* mRNA have been associated with loss of histone H3K27 trimethylation, demethylation of promoter CpGs, and increased global bi-allelic expression from chromosome X consistent with XCI erosion [2, 3, 13]. However, less is known about the gene-wise patterns of XCI in hiPSCs and how individual genes are affected by loss of *XIST* expression. The variation in patterns of de-repression resulting from XCI erosion in hiPSCs can have important implications for biological modeling, for instance, by rescuing or inducing phenotypes associated with deleterious gene variants in X-linked developmental disorders [1].

Here, we describe a systematic analysis of the landscape of XCI in hiPSCs and the gene-wise patterns of de-repression associated with silencing of *XIST*. We find that although hiPSCs had largely uniform patterns of XCI that were shared with human tissues, they displayed considerable line-to-line variation in the number of de-repressed genes and their degree of bi-allelic expression. The patterns of erosion of XCI, associated with the loss of *XIST*, were found to be non-random and resulted primarily from de-repression of specific subset of epigenetically variable genes, including genes that have a degree of escape in human tissues. The XCI erosion in a subset of lines amplified female-biased expression at hypomethylated sites and decreased male-biased differences in X chromosome as well as *XIST*-bound autosomal genes. Importantly, several genes underlying developmental disorders reactivated in the hiPSCs, which could modify biological effects associated with gene variants if not properly accounted for in the study designs. Our results highlight previously unappreciated gene-wise patterns of XCI in hiPSCs and implicate genomic mechanisms driving X-regulated gene expression. The gene-wise variability in escape from XCI needs to be carefully monitored in hiPSC study design, but it can provide novel insights into genetic mechanisms regulated by escape from XCI in humans.

## Results

### hiPSC cultures were clonal and yielded unbiased ASE estimates from bulk RNA-seq

**Fig. 1:**
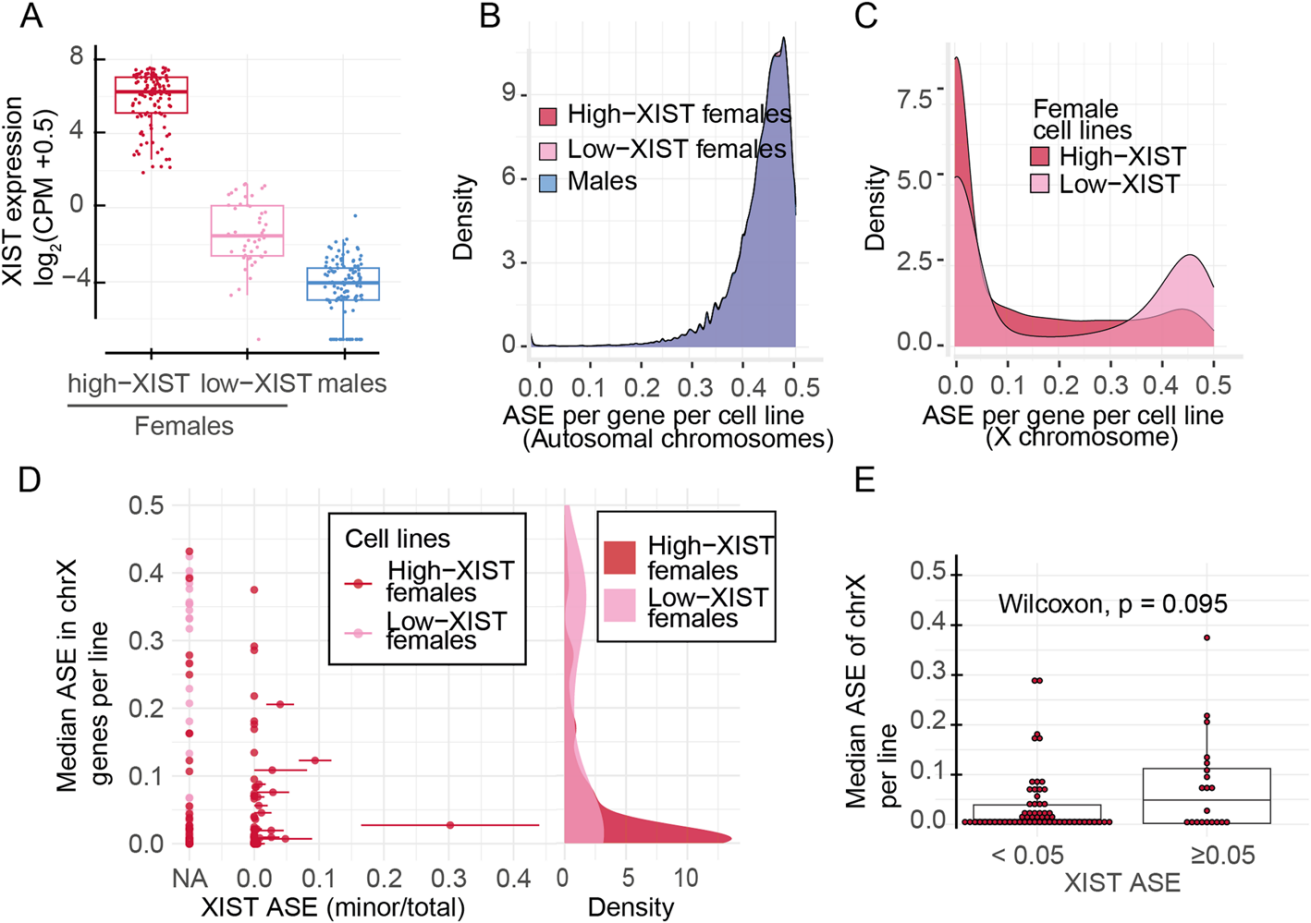
*XIST* expression is associated with de-repression from XCI. **A** The hiPSCs were divided by sex and *XIST* expression to males (*n* = 117) and females with low (*n* = 47) or high *XIST (n* = 118*)* levels. Female hiPSCs were divided into two groups depending on their *XIST* expression levels being greater or smaller than 1.5 log2(CPM + 0.5). **B** The male and female lines with low or high levels of *XIST* have similar ASE in autosomal genes (Kolmogorov–Smirnov test, *p*-value_(lowXIST/highXIST)_ = 0.20, *p*-value_(male/highXIST)_ = 0.22, *p*-value_(male/lowXIST)_ = 0.75; *n* = 12,500 genes in total, *n* = 11,744 genes in males, *n* = 11,039 genes in low-XIST females, *n* = 11,807 genes in high-XIST females and 10,516 genes common in all). **C** Female lines with low *XIST* and high *XIST* levels differ in the ASE distribution of their X chromosome genes, with low-XIST female lines having more bi-allelic expression than high-XIST female lines (*p*-value < 2.2 × 10^–16^, Kolmogorov–Smirnov test, *n* = 411 genes in total, *n* = 344 genes in low-XIST females, *n* = 390 genes in high-XIST females, and *n* = 323 genes common in all female lines). **D** The ASE for *XIST* could be defined for 80 female lines (out of 165). Majority of these lines (*n* = 60) had monoallelic expression (ASE < 0.05, one-sided binomial test, *p*-value < 0.05) of *XIST* consistent with clonality of Xi in the cultures but varied in the level of bi-allelic expression for X-chromosome genes, suggesting variable epigenetic patterns of the female hiPSCs. Error bars indicate 95% confidence intervals for the *XIST* ASE estimates. On the right side, density plots for the median ASE values in two female groups are shown. **E** The 20 lines that had traces of bi-allelic expression of *XIST* (ASE ≥ 0.05) did not differ in median ASE of X-chromosome genes (Wilcoxon rank-sum test, *p*-value = 0.095)

To investigate gene-wise landscape of XCI in hiPSCs, we analyzed allele specific expression (ASE) from RNA sequencing (RNA-seq) data in 282 karyotypically normal cell lines from the HipSci project (165 females, Additional file 2: Table S1) [14]. Unlike human tissues, hiPSC lines are thought to be clonal for Xi enabling assessment of XCI through ASE analysis from bulk RNA-seq data [1, 2, 12]. We computed ASE by calculating the total fraction of RNA-seq reads that mapped to the allele with fewer sequence reads for each gene. In total, ASE was computed for 12,911 genes that had at least 20 reads overlapping heterozygous sites in one or more cell lines (Additional file 2: Table S2, Table S3). Out of these, 411 genes mapped to the X chromosome (Additional file 2: Table S3). The female lines had on average 72 (median) X-linked genes with ASE data (range: 2–102). We then identified the female lines that had lost *XIST* expression, associated with transcriptional reactivation of the Xi [1–3, 13, 15, 16], and would therefore have eroded XCI. We defined a threshold for low *XIST* mRNA level based on the range of expression in female human tissues in the GTEx project [17] (log_2_CPM (*XIST*) < 1.5, Additional file 1: Fig. S1). We identified 47 low *XIST* female lines and 118 lines with appreciable or high expression of *XIST* (Fig. 1A). While there was no difference in the median ASE for the 12,500 autosomal genes between the female lines with high or low level of *XIST* and 117 male lines (median ASE = 0.44 (95% CI: 0.32–0.50), Fig. 1B), the female hiPSCs with low *XIST* level had significantly higher median and mean ASE at X chromosome genes than females with abundant *XIST* (*p*-value_median_ = 4.60 × 10^–3^ and *p*-value_mean_ = 3.40 × 10^–11^, respectively, one-sided Wilcoxon rank-sum test; median ASE (low-XIST lines) = 0.049 [95% CI: 0.008, 0.229]; median ASE (high-XIST lines) = 0.012 [95% CI: 0.007, 0.022]; mean ASE (low-XIST lines) = 0.179 [95% CI: 0.163, 0.223]; mean ASE (high-XIST lines) = 0.101 [95% CI: 0.094, 0.123]), Fig. 1C, Additional file 1: Fig. S2A-B, Additional file 2: Table S1), consistent with erosion of XCI, in line with previous work [1, 3, 15, 16]. Furthermore, the results confirmed that the loss of *XIST* and erosion of XCI had little or no effect on the distribution of ASE in autosomal genes (*p*-value_(lowXIST/highXIST)_ = 0.20, Kolmogorov–Smirnov test, Fig. 1B), as expected.

Small number of female hiPSCs occasionally undergo reactivation of Xi during reprogramming that is followed by a subsequent random re-inactivation of one of the X chromosomes [5, 18]. The resulting non-clonality can bias ASE estimates from bulk RNA-seq data [19]. This is commonly observed for human embryonic stem cells [20, 21] but affects typically less than 10% of cells in hiPSCs [5, 18]. To assess the impact of potential non-clonality for the ASE estimates, we analyzed allelic expression of *XIST*—transcribed solely from the Xi—to identify lines that were chimeric for Xi. We were able to obtain allelic information for *XIST* for 80 of the 165 female lines and found that most of the hiPSCs (75%) had monoallelic expression of *XIST* (ASE < 0.05, one-sided binomial test, *p*-value < 0.05), consistent with a clonal culture (Additional file 2: Table S1). In 20 lines, we observed a fraction of reads from the second allele of *XIST* that could reflect a re-inactivation of the other X chromosome. For all but one of these lines, there was a significant allelic imbalance of *XIST* with over 90% of the reads mapping to only one of the alleles (Fig. 1D), in line with the previous estimates of non-clonality [5, 18]. We chose to include the lines with fraction of bi-allelic *XIST* expression in further analysis to avoid biasing comparison between the low *XIST* lines with undefined *XIST* ASE due to low sequence coverage. The low level of bi-allelic *XIST* expression was not associated with increased median ASE (*p*-value = 0.09; Wilcoxon rank-sum test) or fraction of genes with ASE > 0.1 (*p*-value = 0.27; Wilcoxon rank-sum test) at the X chromosome (Fig. 1E, Additional file 1: Fig. S2C). Together, these data are in line with a single ancestor for each hiPSC line and suggest that the traces of non-clonality in the cultures do not significantly bias the ASE estimates from bulk RNA-seq data.

### De-repression from XCI in hiPSCs affects distinct sets of genes

**Fig. 2:**
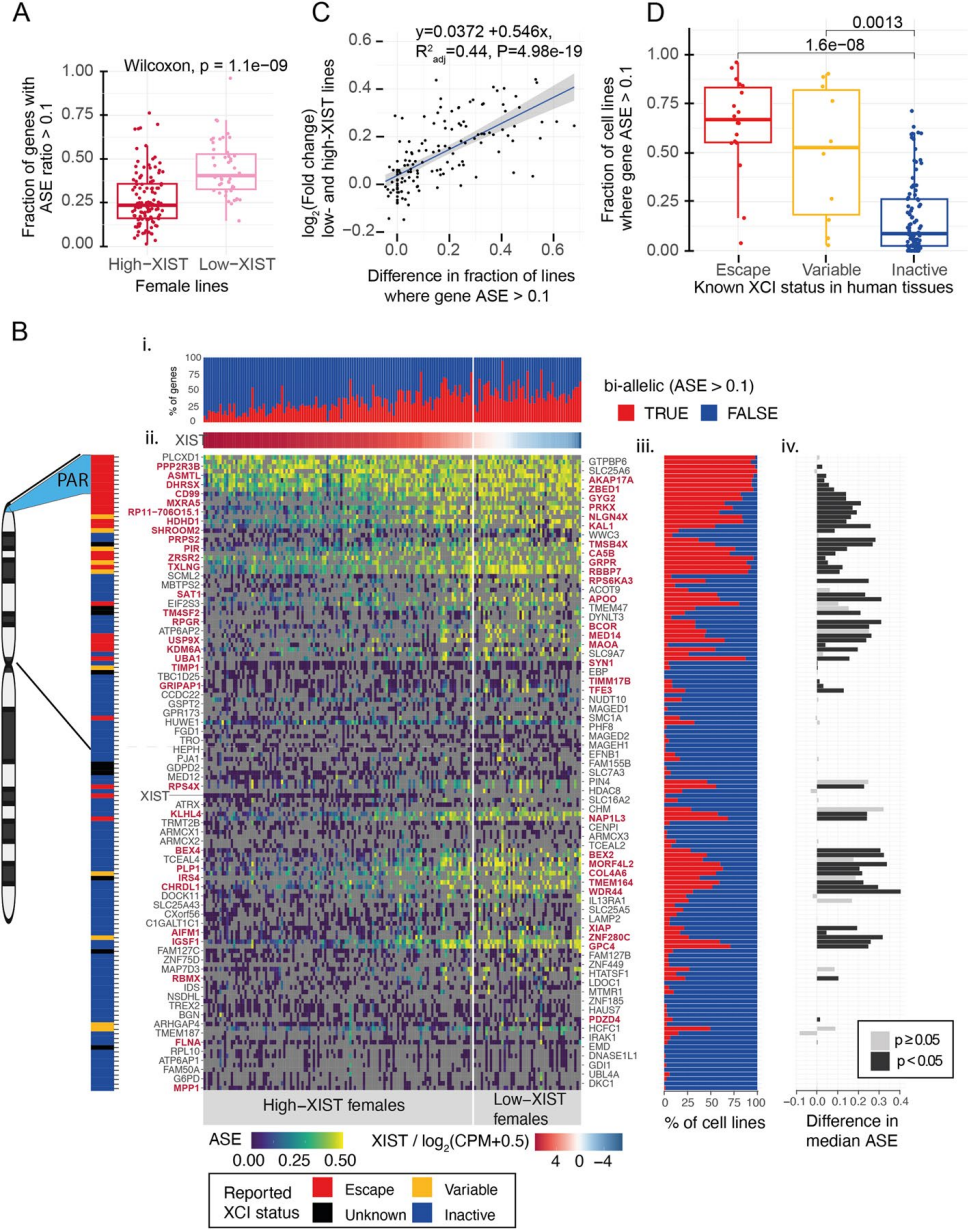
X chromosome ASE landscape in hiPSCs. **A** A Tukey boxplot of the fraction of genes with ASE > 0.1 (one-sided binomial test, FDR *q*-value < 0.01) in female lines with high (*n* = 118) and low (*n* = 47) levels of *XIST* for 139 genes (see Figure S3 panel ii for data of informative genes per line). Female lines with low *XIST* levels had significantly higher fraction of genes with ASE > 0.1 (*p*-value = 1.10 × 10^–9^; one-sided Wilcoxon rank-sum test). **B** Genomic pattern of bi-allelic expression for 139 X chromosome genes in 165 female lines. i a bar graph of the percentage of bi-allelically expressed genes (ASE > 0.1, one-sided binomial test, FDR *q*-value < 0.01) and monoallelically expressed (ASE < 0.1) genes for each line. ii A heatmap of degree of ASE for 139 genes (rows) in 165 female lines (columns). These 139 genes were selected such that there were at least 10 cell lines with available ASE data in all presented genes in both female groups. The genes are oriented according to position in the X chromosome and the 165 lines are in decreasing order of *XIST* expression (*XIST* is highlighted). The low *XIST* lines are separated with a vertical line (white). Cells in gray color indicate missing information. The pseudo autosomal region (PAR) is highlighted in light blue in the chromosome. Names of the genes with significant differences in ASE between low *XIST* and high *XIST* lines (Wilcoxon rank-sum test, *p* < 0.05) are colored in red. The escape status for each gene in humans is color coded on the left bar (escape genes: red, variable: yellow, inactive: blue, black: unknown). iii The percentage of female lines with monoallelic (blue) and bi-allelic (red) expression for each gene is presented as a horizontal bar graph on the right side of the heatmap. iv Difference in the median gene ASEs between the low- and high-*XIST* female lines is presented as a horizontal bar graph. The 58 genes with significant difference in ASE between low and high XIST lines (*p*-value < 0.05, Wilcoxon rank-sum test) are highlighted in black bars. **C** Difference in the fraction of lines where ASE > 0.1 between low *XIST* and high *XIST* female lines is positively correlated with the log fold changes between the gene expression levels of low *XIST* and high *XIST* female lines (*R*^2^ = 0.44, *p*-value = 4.98 × 10^–19^), for 137 genes which had ASE data available in at least 10 cell lines in each of the high-XIST and low-XIST female groups. *XIST* and TM4SF2 (due to missing gene expression data) genes were not included in the plot. **D** Boxplot of fraction of cell lines that have ASE > 0.1 of genes that are known to escape (red), variably escaping (yellow), and inactive (blue) in human tissues. Only the genes which have ASE data in at least ten cell lines in both low-XIST and high-XIST female lines are included, excluding *XIST* and the PAR genes. The fraction of cell lines with ASE > 0.1 is significantly lower for inactive genes than for known escape and variably escaping genes (one-sided Wilcoxon rank-sum test, *p* = 1.60e-08 and 1.30e-03, respectively)

After confirming the clonal XiXa state of the hiPSCs and that we could accurately measure ASE, we sought to better understand the patterns of the XCI in the female lines. Beyond the differences in the mean ASE at X chromosome genes between the low and high *XIST* lines, the ASE distributions were heavily biased toward monoallelic expression (ASE = 0) with long tails and bimodality (Fig. 1C). This suggested that the degree of XCI was varying by cell line and/or by gene in the female lines. To obtain, therefore, a clearer picture of the gene-wise patterns of XCI, we studied ASE of the 411 X chromosome genes that had allelic counts available in at least one of the 165 female lines. We found that 61% of the genes (*n* = 252) had bi-allelic expression in at least one cell line (ASE > 0.1; one-sided binomial test, FDR *q*-value < 0.01, Additional file 2: Table S4). This implied that a large fraction of genes had a potential to, on occasion, escape XCI or become de-repressed in the hiPSCs. To confirm that the high rate of bi-allelic expression was explained by the loss of *XIST* in a subset of lines, we compared the number of genes with ASE > 0.1 (one-sided binomial test, FDR *q*-value < 0.01) between the female lines that had high or low levels of *XIST*. Surprisingly, we observed that many genes had bi-allelic expression in both the high *XIST* and low *XIST* lines. Out of the 252 genes, 209 expressed both alleles in at least one line with high *XIST*, and 211 were bi-allelic in at least one line in the low *XIST* lines (168 were bi-allelic in both, Additional file 2: Table S5). However, while the number of bi-allelically expressed genes in at least one line was similar in the two groups, the total fraction of bi-allelically expressed genes in each line was significantly higher in the female lines with low level of *XIST* than in lines with high *XIST* expression (*p*-value = 1.10 × 10^–9^; one-sided Wilcoxon rank-sum test, high-XIST mean fraction: 0.27; 95% CI [0.244, 0.300], low-XIST mean fraction: 0.44; 95% CI [0.396, 0.488], Fig. 2A, Additional file 1: Fig. S3 panel vi, Additional file 2: Table S1). These results suggested that although lines with low *XIST* level had on average more bi-allelic genes per line, consistent with the erosion of XCI, many of the genes had a propensity to sporadically express both alleles also in lines with high *XIST* levels.

The large number of genes with occasional bi-allelic expression in hiPSCs prompted us to further investigate the genomic patterns of the escape along the X chromosome. In humans and mice, the escape from XCI is tightly regulated by early epigenetic confinement and spatial control of access to the DNA [10, 22]. Further, de-repression, linked with erosion of XCI, has been associated with distinct epigenomic domains [15]. We were, therefore, interested in whether genes at specific loci were more likely to become de-repressed than others. To investigate the genomic patterns of de-repression from XCI, we focused on 139 X chromosome genes for which there were allelic counts from 10 or more lines from both the low and high *XIST* female groups (Fig. 2B, Additional file 1: Fig. S3). This allowed us to analyze the likelihood of de-repression for each gene across multiple female lines. Focusing on the 139 genes, the median number of genes with ASE data per cell line was 59 (range: 2–79). Out of the 139 genes, the majority (122) were bi-allelically expressed at least once in the 165 lines (ASE > 0.1, one-sided binomial test, FDR *q*-value < 0.01). However, both the fraction of genes with bi-allelic expression in each line (1.8–95% of all genes with ASE data per line), as well as the frequency of lines with bi-allelic expression for each gene were highly variable (0–100%, Fig. 2B, panels i and iii). Notably, however, most genes were bi-allelic in only a few lines. Out of the 139 genes, 60 expressed both copies in more than 25% of the lines and 39 genes were bi-allelic in over 50% of the lines. This indicated that although hiPSCs displayed considerable heterogeneity in the escape and de-repression of individual genes, the likelihood for a gene to become de-repressed in hiPSCs was non-random, as some genes were more likely to reactivate than others in line with previous reports [15].

The non-random pattern of de-repression was also clear in the female lines that had low *XIST* level. We identified 41 genes that had significantly higher fraction of cell lines with bi-allelic expression (*p*-value < 0.05, Fisher’s exact test) in the low *XIST* female lines than in the high *XIST* lines (Additional file 2: Table S5, Additional file 1: Fig. S3, panel vi), suggesting that de-repression due to the loss of *XIST* was concentrated to a subset of genes. Escape from XCI typically leads to higher gene expression. Therefore, to confirm that the increased ASE in the female lines with low level of *XIST* resulted from excess de-repression from XCI, we analyzed differential expression of the 139 X chromosome genes between the two groups of female lines (Additional file 1: Fig. S4, Additional file 2: Table S6). We found that the genes that were de-repressed more frequently in low *XIST* lines than in high *XIST* lines commonly also presented with significantly higher gene expression in the low *XIST* female lines (*R*^2^ = 0.44, *p*-value = 4.98 × 10^–19^, Fig. 2C, Additional file 1: Fig. S4). Out of 41 genes that had significantly higher fraction of cell lines with bi-allelic expression (ASE > 0.1, one-sided binomial test, FDR *q*-value < 0.01), 34 were also more highly expressed in low *XIST* lines than in high *XIST* lines (*p*-value = 8.91 × 10^–7^; Fisher’s exact test). Taken together, these findings implied that some genes readily reactivate in the hiPSCs while others remain resistant to the erosion of XCI.

### Loss of  *XIST* is associated with higher degree of de-repression at genes that escape in humans

We next compared whether the genes that were frequently de-repressed or escaped in hiPSCs clustered to any specific chromosomal regions or escaped in human tissues [10]. Expectedly, we observed a high frequency of bi-allelic expression at the pseudo autosomal region 1 (PAR1) in the tip of the p-arm of chromosome X in all lines, and there was no difference in frequency of bi-allelic expression between the high and low *XIST* females (Fig. 2B, Additional file 1: Fig. S3). Beyond genes in the PAR1, genes that are known to escape or that have variable escape in some human tissues were bi-allelically expressed significantly more frequently in the hiPSCs than genes that do not escape in humans (*p*-value = 1.60 × 10^–8^, *p*-value = 1.30 × 10^–3^, respectively; one-sided Wilcoxon rank-sum test, Fig. 2D). The degree of bi-allelic expression was frequently increased for the variable and escape genes with de-repression in the low *XIST* lines, consistent with that escape rarely results in full expression from Xi in humans [10]. Overall, we found 58 genes with significantly higher ASE (*p*-value < 0.05, Wilcoxon rank-sum test) in the females with low *XIST* level than in the high *XIST* group (Fig. 2B, panel iv, Additional file 2: Table S5) including genes that were found frequently bi-allelically expressed also in the high *XIST* lines and the known escape genes. In addition, we observed clusters of genes, such as *GRPR, TXLNG, RBBP7* and *DYNLT3, RPGR,* and *BCOR,* that are inactive in humans but were frequently bi-allellic in the hiPSCs. Recently, deleterious mutations in *BCOR* were associated with reduced differentiation capacity in pluripotent stem cells [23]. Taken together, the frequent bi-allelic expression of human inactive genes in high *XIST* lines could indicate stem cell specific escape of these genes rather than de-repression, suggesting that some epigenetic marks underlying XCI may be subject to removal during reprogramming, and they may have roles in the stem cell state and differentiation capacity of the hiPSCs.

Finally, we compared the patterns of XCI de-repression between the female lines with high or low levels of *XIST*. The de-repression associated with the loss of *XIST* clustered to regions of consecutive genes in the p- and q-arms of chromosome X (Fig. 2B). In contrast, genes at other regions were de-repressed in only a handful of lines. This implied that some regions were particularly sensitive to the loss of *XIST.* Remarkably, the genes in these regions were more likely bi-allelic also in female hiPSCs with high *XIST* and these overlapped with known escape genes in humans. However, the female lines with low *XIST* had even higher frequency of de-repression of the known escape and variable genes, as well as for the inactive genes than the high *XIST* females (*p*-value = 1.49 × 10^–8^ for escape and variable genes, *p*-value = 4.64 × 10^–13^ for the inactive genes; one-sided paired Wilcoxon rank-sum test, Additional file 1: Fig. S2D).

### Erosion of XCI takes place in cultures with appreciable *XIST* expression.

**Fig. 3:**
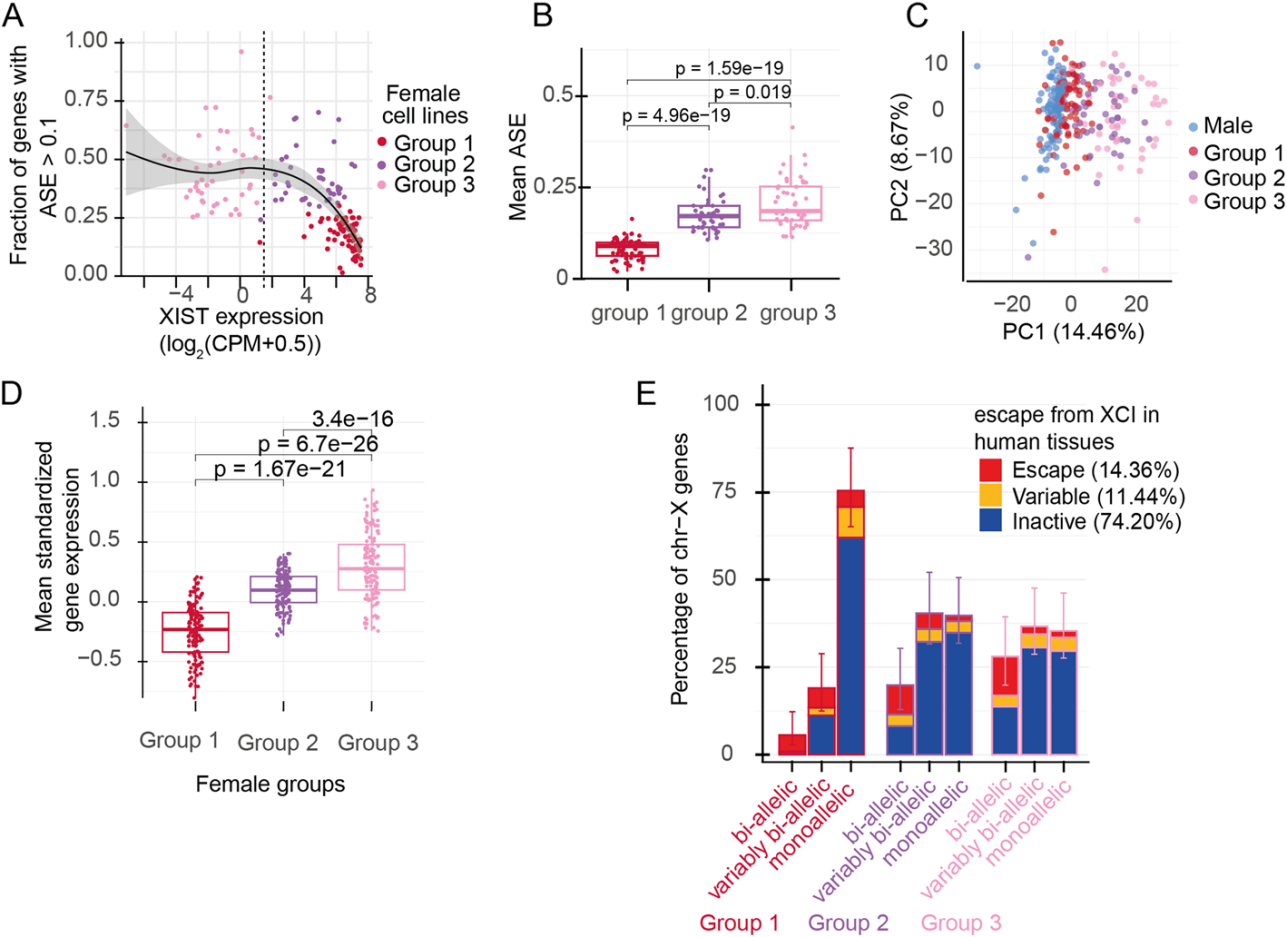
The *XIST* expression and fraction of bi-allelic genes defines three groups of female lines. **A** The relationship of *XIST* expression and fraction of X chromosome genes with bi-allelic expression (ASE > 0.1, one-sided binomial test, FDR *q*-value < 0.01). K-means clustering of *XIST* level and fraction of genes with ASE > 0.1 identifies three groups of female lines. group 1: high-*XIST* female lines with low level of bi-allelic expression (red, *n* = 74), group 2: intermediate female lines that express *XIST* but have high fraction of de-repressed genes (violet, *n* = 45). Group 3: low-*XIST* females with high fraction of de-repressed genes (pink, *n* = 46). The vertical dashed line indicates the threshold for XIST expression level which was used to define low- and high-XIST female groups earlier. **B** Mean ASE of X-chromosome genes is significantly different between the different groups of female lines with the highest mean ASE in group 3 female lines (Wilcoxon rank-sum test; group 1 – group 2: *p*-value = 4.96 × 10^–19^; group 2 – group 3: *p*-value = 1.9 × 10^–2^, group 1 – group 3: *p*-value = 1.59 × 10^–19^). **C** The first two principal components of PCA for X chromosome gene expression (percentages of variance explained by each principal component are shown inside parentheses). The lines are colored by sex and the female XCI stage (male blue, females in group 1: red, group 2: violet, group 3: pink). **D** Tukey style boxplots of the mean standardized gene expression levels (Z-score) in three female groups for the X chromosome genes whose individual standardized expressions are shown in Additional file 1: Fig. S4. *XIST* has been excluded in the boxplots. Gene expression increased for X chromosome genes with more bi-allelic expression (paired *t*-test, group 1 – group 2: *p*-value = 1.67 × 10^–21^, group 2 – group 3: *p*-value = 3.40 × 10^–16^, group 3 – group 1: *p*-value = 6.70 × 10^–26^). **E** Percentage of escape, variable, and inactive genes in human tissues (Table S1 from [10]) with bi-allelic (> 80% of lines with ASE > 0.1), variably bi-allelic (< 80% of lines with ASE > 0.1), or monoallelic (ASE < 0.1) expression in hiPSCs. Group 1 female hiPSCs had similar percentage of inactive and variably bi-allelic genes (at least one line with ASE > 0.1), and slightly less bi-allelic genes (> 80% of lines with ASE > 0.1) than reported for human tissues (human percentages shown in figure legend) (Chi-squared test for equality of proportions, *p*-value = 4.25 × 10^–5^). The group 2 and group 3 female lines had significantly higher fraction of bi-allelic and variably bi-allelic genes than in human tissues (Chi-squared test for equality of proportions, *p*-value = 1.12 × 10^–20^ and *p*-value = 4.12 × 10^–22^, respectively). In each of the female group comparisons with human tissues, only the genes that had available information in both groups were included

Low level of *XIST* mRNA is typically used as a marker for XCI erosion in female hiPSCs. The observed line-to-line variability in de-repression led us to further investigate the relationship between the *XIST* expression and the XCI erosion in the female lines. Fitting a LOWESS regression curve for the *XIST* level and the fraction of X-chromosome genes that had ASE > 0.1 revealed that the *XIST* expression had an inverse linear relationship with the ASE, but only in lines where *XIST* mRNA was still abundantly present (Fig. 3A). However, the relationship with *XIST* expression and fraction of genes escaping plateaued before the loss of *XIST*. The mean ASE of the X chromosome was strongly correlated with the fraction of genes with ASE > 0.1 (*r*^2^ = 0.96) and followed a similar inverse relationship with *XIST* levels in lines where *XIST* was abundantly present (Additional file 1: Fig. S5A). Fitting a sigmoid curve for *XIST* expression and mean ASE suggested a mid-point for the linear change (inflection point) at 6.6 logCPM of *XIST* expression (with a slope of − 0.78) that plateaued at mean ASE of 0.2 (the asymptote, Additional file 1: Fig. S5B). This supported previous findings [15] that although low *XIST* expression is informative of the XCI erosion in hiPSCs, the erosion takes place in the cells in the culture at stages where *XIST* expression is not yet exclusively indicative of the erosion.

The non-linear relationship of *XIST* expression and XCI erosion motivated us to define the state of XCI in female lines by combining information from *XIST* expression and the fraction of de-repressed genes in each line. Therefore, we performed k-means clustering by using *XIST* mRNA levels and fractions of genes with ASE > 0.1. The optimal number of clusters was determined as three by the gap statistic method [24] that revealed three distinct patterns of XCI (Fig. 3A). Firstly, a group with high expression of *XIST* and low level of de-repression (0.18 median fraction of genes with ASE > 0.1) separated from the rest of the lines (high *XIST* group / group 1, *n* = 74). The remaining lines clustered into two groups that had a significantly higher fraction of genes with ASE > 0.1 than the group 1 female lines. But while group 2 lines had an appreciable *XIST* expression (group 2 / intermediate group, 0.39 median fraction of genes with ASE < 0.1, *n* = 45), group 3 had a low level of *XIST* (0.41 median fraction of genes with ASE > 0.1, *n* = 46) and corresponded to the original threshold for loss of *XIST* defined in the beginning of the study (Additional file 1: Fig. S5C). Although group 2 and group 3 had a similar fraction of genes with ASE > 0.1 (*p*-value = 0.32; Wilcoxon rank-sum test), the overall degree of de-repression was significantly higher in the group 3 than in the intermediate group 2 lines (*p*-value = 1.90 × 10^–2^; Wilcoxon rank-sum test, Fig. 3B, Additional file 2: Table S1), suggesting progressive reactivation of Xi in the cultures. Notably, however, the difference in the mean ASE was much greater between group 1 and group 2, in line with the non-linear relationship with *XIST* expression. Similarly, we observed no association between the *XIST* levels and the mean ASE within each female group (Additional file 1: Fig. S5D). A progressive transcriptional reactivation was visible in the first principal component of principal component analysis (PCA) of X chromosome gene expression, but not in autosomes, capturing 14.5% of variance (Fig. 3C,D, Additional file 1: Fig. S5F). We compared the percentage of genes that were monoallelic (no cell lines with bi-allelic expression), bi-allelically expressed in > 80% of lines (bi-allelic genes), and that were variably bi-allelic (< 80% of lines with bi-allelic expression) in hiPSCs to the percentages of escape, inactive, and variably escaping genes observed in human female tissues [10]. The comparison revealed similar fractions of variable and inactive genes and slightly smaller fraction of bi-allelic genes in the group 1 hiPSCs and human tissues (Chi-squared test for equality of proportions, *p*-value = 4.25 × 10^–5^, Fig. 3E, Additional file 1: Fig. S2E). This showed that the bi-allelic expression was overall increased with XCI erosion in all lines with large overlap to known escape genes. The group 1 lines had the most intact XCI with the highest similarity to humans. In comparison, the group 2 and group 3 lines had significantly higher fraction of variably bi-allelic genes, likely resulting from progressive erosion of XCI (Chi-squared test for equality of proportions, *p*-value = 1.12 × 10^–20^ and *p*-value = 4.12 × 10^–22^, respectively).

### The erosion of XCI induces the expression of genes with female-biased expression

**Fig. 4:**
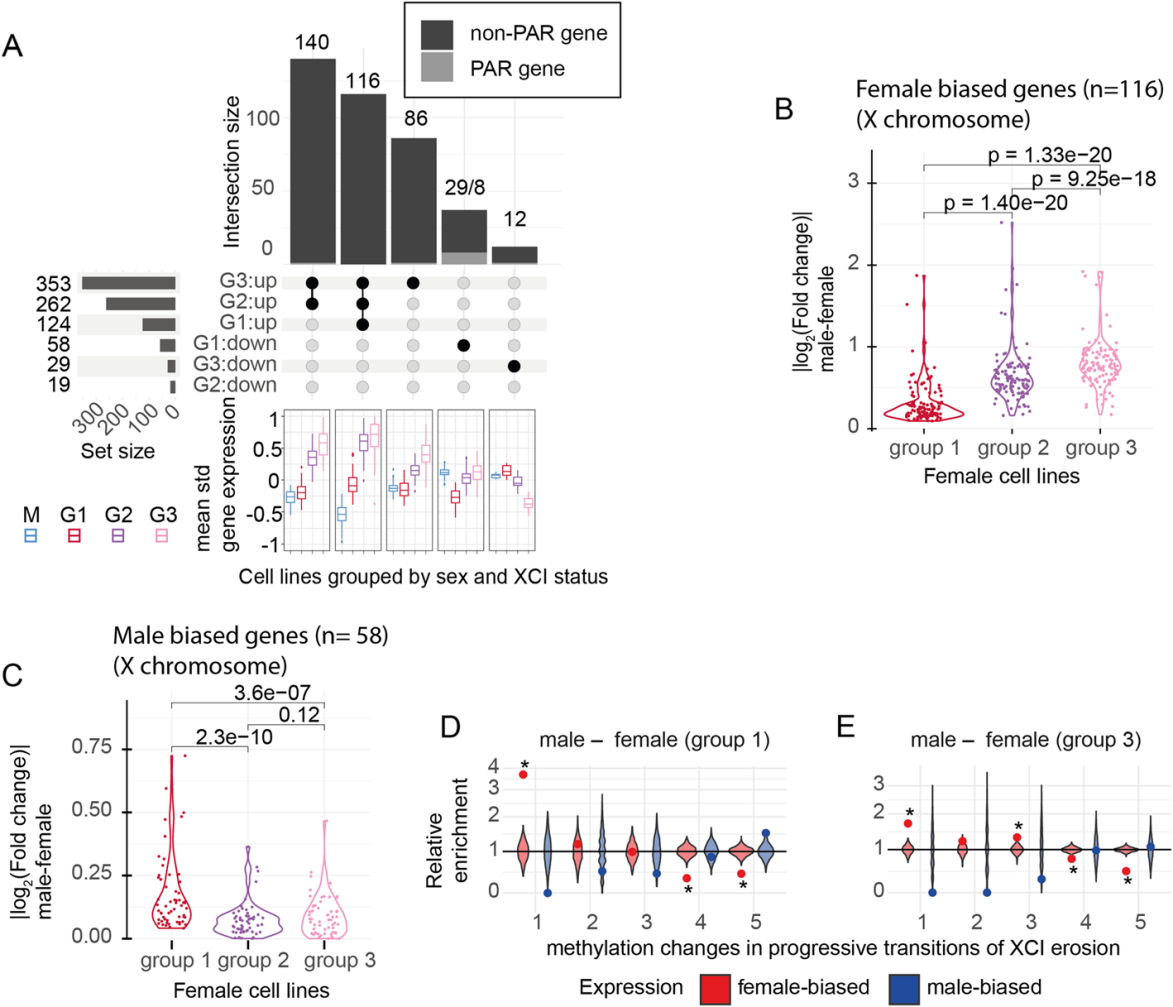
Erosion from XCI enhances female-biased expression and reduces male-biased expression in the X chromosome. **A** An upset plot of the overlap of significantly differentially expressed X-chromosomal genes between male lines and the group 1 (G1), group 2 (G2), and group 3 (G3) female lines with different directions of effects in the female groups. The upset plot is cropped to display the 5 largest overlapping gene groups (excluding 45 genes). The full plot is displayed in Figure S5G. The horizontal bar graph displays the total number of female- and male-biased genes for each group. Boxplots below the upset plot represent the mean standardized expression for males (M), and the group 1 (G1), group 2 (G2), and group 3 (G3) female lines for the subsets of overlapping genes with significant male–female differences in expression. **B** Violin plot of absolute log_2_(fold changes) for X chromosome genes with female-biased expression in all three female groups (*n* = 116 genes, excluding *XIST*). The absolute log_2_(fold changes) increase significantly between group 1 and group 2 (paired Wilcoxon rank-sum test, *p*-value = 1.40 × 10^–20^), group 2 and group 3 (paired Wilcoxon rank-sum test, *p*-value = 9.25 × 10^–18^) and group 1–group 3 (paired Wilcoxon rank-sum test, *p*-value = 1.33 × 10^–20^). **C** A violin plot of absolute log_2_(fold changes) for X-chromosome genes with male-biased expression in group 1 (*n* = 58, including genes in cropped out categories). The absolute log_2_(fold changes) decrease significantly between group 1 and group 2 (paired Wilcoxon rank-sum test, *p*-value = 2.30 × 10^–10^), group 1 and group 3 (paired Wilcoxon rank-sum test, *p*-value = 3.60 × 10^–7^). There was no significant difference between group 2 and group 3 (paired Wilcoxon rank-sum test, *p*-value = 0.12). **D**, **E** Overlap of male- and female-biased genes for group 1 (**D**) and group 3 (**E**) with five (1–5) consecutive hypomethylation changes associated with XCI erosion [25]. A relative enrichment of differentially expressed genes to hypomethylated sites was tested with hypergeometric test (colored points) and by permutation with 1000 random gene sets (violin plots). Significant enrichment (adjusted *p*-value < 0.05) is noted by asterisk

Female hiPSCs with low levels of *XIST* have been shown to differ in gene expression from the female lines with abundant expression of *XIST* [3, 5, 16]. How variability in the de-repression of inactive genes affects male–female differences has been less studied, however. To investigate how de-repression affects sex differences in gene expression in the X chromosome, we compared the 3 groups of female lines to 117 male hiPSCs (Additional file 2: Table S6). The analysis revealed a progressive, general upregulation of X chromosome genes in the group 2 and 3 female lines that had increased levels of de-repressed genes (Fig. 4A, horizontal bars, Additional file 1: Fig. S5G). This was also evident from a growing number of increasingly inclusive sets of X chromosome genes that had higher expression in the three groups of female lines than in males. We identified 124 upregulated genes in the high *XIST* group 1, 262 in the intermediate group 2, and 353 in the low *XIST* group 3 lines (Fig. 4A). Close to all upregulated genes in the group females were found significantly upregulated also in the group 2 and group 3 females (116 out of 124 genes, 94%, adjusted *p*-value < 0.05). Similarly, of the 262 significantly upregulated genes in group 2, 97% (256 genes) were upregulated also in group 3 females. Notably, the mean expression of the upregulated genes that overlapped between the three female groups increased in the lines with a higher degree of de-repression. This resulted in significantly greater differences between males and the group 2 and group 3 female lines than between males and the group 1 females (Fig. 4B). Conversely, genes that had significantly lower expression in the group 1 female lines than in male hiPSCs mostly had attenuated sex bias in the group 2 and group 3 females (Fig. 4C). Out of the 58 downregulated genes in group 1 females, 37 genes were significantly different from males only in group 1 female lines. These included PAR genes with a known male-biased expression in humans (Fig. 4A). Furthermore, we found 11 genes that had significantly lower expression in group 1 females than in males but were upregulated in group 3 female lines. Two of these (*CLCN4* and *MID1*) were PAR genes and the rest spread the p-arm (*SCML1, DMD, PLP2, PRICKLE3, USP27X, SHROOM4*) and q-arm (*EFNB1, HMGB3, L1CAM*) of X, suggesting another mechanism for the flipping of the male-biased expression for these genes in the group 3 lines. In summary, the findings demonstrated that increased de-repression with erosion of XCI results in the upregulation of X chromosome genes. This resulted in magnified sex differences in the expression of genes that had female-biased expression in group 1 hiPSCs and decreased effects of genes with male-biased expression.

### De-repression of X chromosome takes place at domains with repressive chromatin marks and erosion-associated hypomethylation

We explored the genomic mechanism driving the observed de-repression of X chromosome expression at different stages of XCI. The transcriptional reactivation during XCI erosion is associated with heterochromatin remodeling at domains defined by repressive H3K27 trimethylation (H3K27me3), while heterochromatin H3K9me3 domains are unaffected [15]. To further confirm that the increase in X chromosome transcription was associated with de-repression of X, we analyzed differences in the expression of X-linked genes between the three female groups and studied their overlap with previously published repressive histone marks from human embryonic stem cells with either inactive or eroded X chromosome [15]. The analysis yielded 266 and 382 differentially expressed genes between group 1 and groups 2 and 3, respectively (Additional file 2: Table S6). A comparison with H3K27me3 and H3K9me3 histone marks of the X chromosomes in XaXi and XaXe cells revealed significant enrichment for upregulated X-linked genes in group 2 and group 3 at regions with H3K27me3 marks in inactive XaXi stem cells, but not for H3K9me3 (group 2 *p*-value = 4.9 × 10^–7^, group 3 *p*-value = 4.5 × 10^–9^, for H3K27me3, hypergeometric test, Additional file 1: Fig. S6A,B), consistent with XCI erosion. By comparison, repressive histone marks of eroded lines XaXe showed no enrichment with the differentially expressed genes.

Erosion of XCI has been reported to lead progressively to a global hypomethylation in iPSCs [25]. We, therefore, analyzed the overlap of the genes with male-biased and female-biased expression in the different female groups with changes in methylation states that were previously identified to take place in five consecutive transitions (1–5) between progressive stages of XCI erosion (Additional file 1: Fig. S6C) [25]. Overall, 10–20% of female biased X-linked genes overlapped with hypomethylated sites (Additional file 1: Fig. S6E). The largest overlap of female biased genes in group 1 and group 2 were with hypomethylation at the first transition (38% and 24%) and for group 3 with hypomethylation at transition 4 (22%). The female-biased X-linked genes in all female groups were significantly enriched for hypomethylation associated with a transition at an early stage of XCI erosion (transition 1, group 1 *p*-value = 3.7 × 10^–23^, group 3 *p*-value = 2.1 × 10^–14^, hypergeometric test, Fig. 4D,E and Additional file 1: Fig. S6D). For further validation, we studied the magnitude of the enrichment with 1000 same-sized random gene sets that confirmed the enrichment for the female-biased genes at the early hypomethylated sites (Fig. 4D,E and Additional file 1: Fig. S6D). We observed a trend for enrichment of hypomethylation associated with the consecutive transitions 2 and 3 for genes with female-biased expression in group 2 and group 3, consistent with progressive erosion of XCI in the female groups. Conversely, we observed that up to 50% of the identified male-biased genes overlapped with regions that are hypomethylated at late stages of XCI erosion (transitions 4 and 5) but had little (< 5%) or no overlap with hypomethylated sites corresponding to the early stages of erosion (transitions 1–3, Additional file 1: Fig. S6E), highlighting the progressive mechanism driving the loss of male-biased gene expression in erosion.

### XCI erosion is associated with dose-dependent sex differences in gene expression at hypermethylated sites in autosomes

**Fig. 5:**
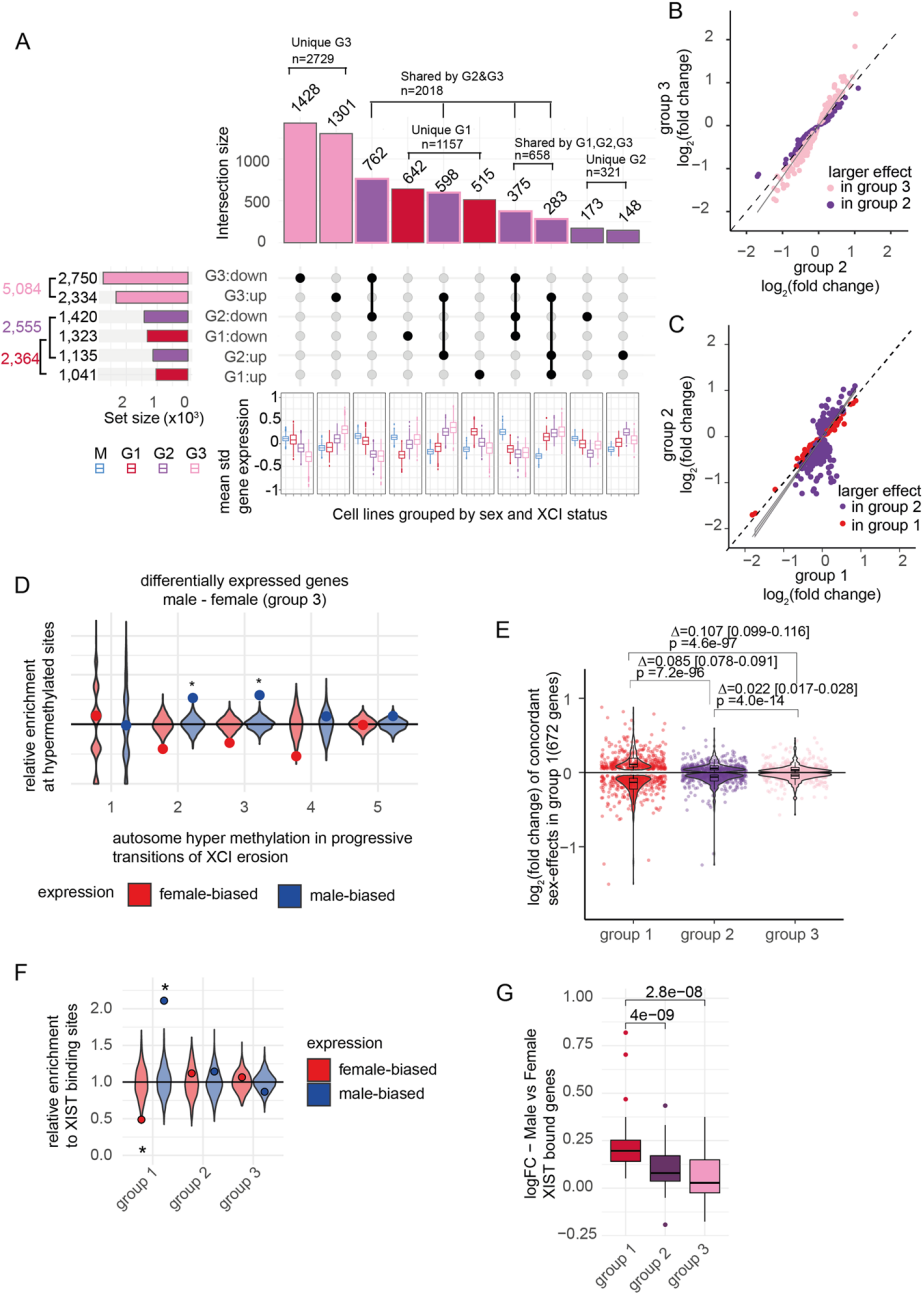
XCI erosion drives sex differences in autosomal genes enriched for hypermethylation and *XIST*-binding regions. **A** An upset plot for autosomal genes that were found significantly differentially expressed between males and the three groups of female lines. The upset plot is cropped to display the 10 largest overlapping gene groups (excluding 549 genes). The full plot is displayed in Additional file 1: Fig. S7A. The horizontal bar graph displays the total number of female- and male-biased genes for each group. The horizontal bars are colored by the female group: pink for group 3, violet for group 2, and red for group 1. The boxplots below the upset plot present the mean standardized gene expression for male lines (M) and the group 1 (G1), group 2 (G2), and group 3 (G3) female lines for subsets of overlapping genes. The vertical bars indicate the sharing of significant male–female differences between the female groups. The unique effects in group 3 are colored in pink, the effects shared by group 2 and group 3 are colored in violet and outlined with pink, the unique effects in group 2 are colored in violet without outline, and the effects that are unique for group 1 are colored in red. The non-concordant effects are cropped out (see Additional file 1: Fig. A). **B**, **C** A scatter plot of log (fold-changes) in group 2 and 3 (**B**), and group 2 and 1 (**C**) for the 2018 differentially expressed genes from males that are shared between group 2 and 3. The genes are colored by the group with larger effect (pink in group 3, violet group 2, red in group 1). The effect sizes are the largest in group 3 (below the diagonal for downregulated genes and above the diagonal for upregulated genes) and weakest in group 1 in line with a dose-dependent effect of X dosage on autosomal expression. A diagonal line (dashed) and regression line for fold-changes are shown in the plots. **D** A relative enrichment of female- and male-biased genes (red, blue, respectively) in group 3 for hypermethylation changes at autosomes during progressive transitions in XCI erosion (1–5). Male-biased genes in group 3 are enriched for hypermethylation (adjusted *p*-value < 0.05, denoted with asterisk). Overlap with 1000 random gene sets are shown as violin plots. **E** A violin plot and embedded Tukey style boxplot of 672 genes significant in group 1 and same direction of effect in other groups. The differences in absolute log_2_ (fold-changes) are shown above plots with associated *p*-values, paired *t*-test (*p*-value (group 3 – group 2) = 4.00 × 10^–14^, *p*-value (group 3 – group 1) = 4.60 × 10^–97^, *p*-value (group 2 – group 1) = 7.20 × 10^–96^). **F** A relative enrichment of female- and male-biased genes (red, blue respectively) for *XIST* binding sites [26] at autosomes in different female groups. Male-biased genes are significantly enriched for XIST binding in autosomes (adjusted *p*-value < 0.05, denoted with asterisk). Overlap with 1000 random gene sets are shown in violin plots. **G** Log fold change of male–female difference for 55 *XIST*-bound autosomal genes in the female groups 1–3. The male bias is diluted with reduced expression of *XIST* for group 2 (paired Wilcoxon test, *p* = 4e-09) and group 3 (paired Wilcoxon test, *p* = 2.8e-08) compared to group 1 with high XIST levels

In autosomes, differential gene expression analysis between male and the three groups of female lines revealed increasing numbers and magnitudes of sex differences associated with the progressive stages of XCI erosion. We observed twice the number of significant genes in the group 3 females (*n* = 5084 genes with adjusted *p*-value < 0.05) than in the group 1 (*n* = 2364) or in the intermediate ASE group 2 (*n* = 2555) compared to males (Fig. 5A, Additional file 1: Fig. S7A, pink, red, and violet horizontal bars, respectively). The higher number of significant genes in group 3 was unrelated to differences in power due to variable sample sizes: group 1 had the largest sample size (*n* = 74) and the lowest number of differentially expressed genes, and groups 2 and 3 were equally sized (*n* = 45 and 46, respectively). Instead, over half (54%) of the 5084 significant genes were exclusively identified in group 3 (*n* = 2729 genes, 1428 downregulated, and 1301 upregulated, Fig. 5A, pink vertical bars). The rest were nearly completely shared by the intermediate group 2 females (86%, 2018 out of 2355 genes, Fig. 5A, pink shaded, violet bars), and they included 79% of the sex differences that were observed in group 2, consistent with progressive erosion of XCI. Remarkably, 95% of the 2729 genes exclusively significant in group 3 females had the same direction of effect (albeit not significant) in group 2 (*n* = 2581 genes) but had only modestly higher than expected overlap with group 1 (56%, 1.1-fold enrichment to 50% *p*-value = 4.5 × 10^–5^), suggesting induced novel autosomal sex differences in the group 2 and group 3 lines by the de-repression of X.

The increasingly inclusive sets of concordantly differentially expressed genes in group 2 and group 3 associated dose-dependently with the de-repression of XCI, so that the mean expression in group 2 was intermediate to those in group 3 and group 1 for large fraction of genes (Fig. 5A boxplots). The magnitude of difference to males was on average significantly smaller in group 2 than in group 3 for the 2018 shared genes (difference = 0.034, 95% CI: 0.023–0.045, *p*-value = 1.9 × 10^–9^, *t*-test) with 70% of the genes (*n* = 1404) having a greater effect in group 3 (Fig. 5B,C and Additional file 1: Fig. S7B). Moreover, only few genes had the highest or lowest expression and were uniquely significantly different from males in group 2 (173 downregulated, 148 upregulated), together implying that autosomal gene expression was directly regulated by X chromosome dosage. Erosion of XCI is associated with global methylation changes in iPSCs [25]. This prompted us to study whether the sex differences in gene expression were enriched for previously reported sites with changes in methylation in autosomes [25]. While there was no enrichment for differentially expressed genes at hypomethylated sites (Additional file 1: Fig. S7F, Fig. S6F), we found that a small fraction (< 5%) of the male-biased genes in group 3 females overlapped with the hypermethylated sites and were significantly enriched for sites of hyper-methylation characteristic of XCI erosion transitions 2 and 3 (transition 2 *p*-value = 3.5 × 10^–4^, transition 3 *p*-value = 2.5 × 10^–7^, Fig. 5D, Additional file 1: Fig. S7E, Fig, S6G).

### Male-biased expression is associated with *XIST*-binding at autosomal genes in group 1 lines.

In comparison to group 2 and 3, the sex differences in group 1 lines with high *XIST* level had less overlap with the others: Only 658 (33%) of the 2018 common genes between group 2 and 3 were significantly different between males and the group 1 females. Instead, we identified 1157 genes that were uniquely changed only in the group 1 females, comprising 49% of the sex effects in group 1 (Fig. 5A, red colored vertical bars). Out of these, 672 genes had the same direction of effect in all groups. Conversely to the increasing magnitudes of female-biased expression associated with differentially expressed genes in group 2 and group 3, the group 1 genes followed an opposite dose-dependent trend with the lowest fold changes and the smallest overlap of same-direction of effects in group 3 (Fig. 5E). This implied that in addition to novel sex differences that emerged with higher degree of de-repression, some male–female differences observed in lines with high *XIST* level faded out with erosion of XCI.

Recently, *XIST* was shown to directly regulate the expression of autosomal genes *in trans* in naïve stem cells through diffused binding to autosomes [26]. This led us to hypothesize that the differences in autosomal gene expression in the eroding female iPSC lines was driven by the reduced binding of *XIST* mRNA at autosomal genes leading to the de-repression of these genes. Excitingly, we found that genes with male-biased expression in group 1 were enriched for sites with *XIST* binding in XaXi cells (55 out of 1323 genes, relative enrichment = 2.11, *p*-value = 8.7 × 10^–8^, hypergeometric test, Fig. 5F), but no enrichment was seen for the eroded groups. This implicated that the depletion of *XIST* expression in XCI erosion was directly driving differences in male-biased autosomal gene expression. For these 55 *XIST*-bound genes, we observed on average larger differences in expression than for the rest of the male-biased genes (logFC = 0.196 (95% CI: [0.155, 0.237] vs. logFC = 0.106 (95% CI: [0.100, 0.111], *p*-value = 7.9 × 10^–8^, Willcoxon rank-sum test, Additional File 1: Fig. S7D), and there was a decline in the magnitudes of differences to males in female lines with reduced levels of *XIST*, implicating a direct mechanism of X-regulated gene expression changes in the autosomes (Fig. 5G).

### XCI erosion associates with reduced differentiation ability and gene expression changes in developmental processes

Previous work has shown that XCI erosion hampers the differentiation ability of stem cells [27, 28]. To test this, we studied endodermal differentiation ability, previously recorded from the same lines [23]. We found that the group 2 and group 3 female lines had slightly lower differentiation ability than the group 1 females. However, there was substantial line-to-line variability suggesting that alternative factors contribute to the lines’ differentiation ability besides XCI status (Additional file 1: Fig. S7C). Further, we found no differences in the expression of pluripotency markers (OCT4/POU5F, SOX2, NANOG, Additional file 2: Table S6), suggesting the erosion stages in these lines were not substantially altering pluripotency properties of the studied cells.

To explore whether some specific biological functions were affected by the de-repression of XCI, we performed gene ontology (GO) enrichment analysis for sex differences detected in the low XIST group 3. We observed no enrichment for specific biological processes for the upregulated genes, suggesting that the affected genes play roles in many different pathways and biological processes. The genes downregulated in the de-repressed group 3 and group 2 lines were enriched for diverse developmental pathways and included functions related to chromosome segregation (Additional file 2: Table S7), highlighting the potential impact of the erosion to these functions.

### Genes underlying X-linked developmental disorders vary in de-repression in hiPSCs

**Fig. 6:**
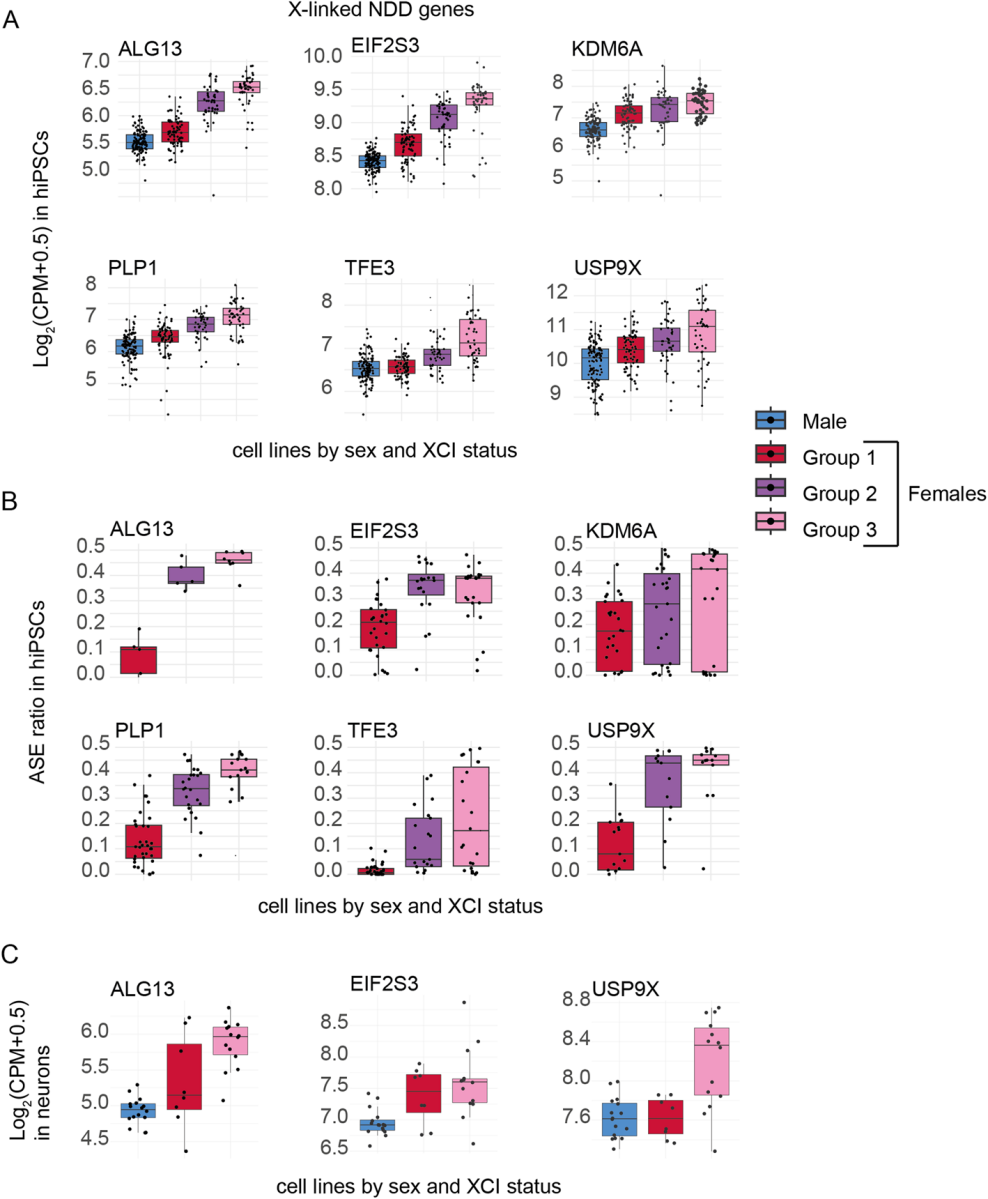
Variable escape from XCI in the hiPSCs masks dominant loss of function effects of genes in X-linked neurodevelopmental disorders (NDD). **A** Boxplots of expression of selected genes linked with X-linked NDD with ASE and differential expression data that have significantly induced expression in group 3 female lines. **B** Boxplots of median ASE for the disease genes show higher ASE in group 3 female lines. **C** Boxplots of gene expression in differentiated neurons with high or low expression of *XIST* differentiated from the same hiPSCs. Boxplots are in Tukey style

Previously, erosion of XCI has been reported to mask genetic effects related to X-linked developmental disorders [1]. This led us to systematically explore how line-to-line variability in escape from XCI impacts the expression of genes where gene disabling variants have been previously implicated in neurodevelopmental disorders (NDD) both in X chromosome and autosomes. We gathered 458 genes previously associated in neurodevelopmental disorders (NDD) [29–35]. We identified 78, 114, and 184 NDD genes that were significantly (adjusted *p*-value < 0.05) differentially expressed in the group 1, group 2, and group 3 females compared to males, respectively (Additional file 2: Table S6). A likely primary genetic mechanism for a dominant loss of function (LoF) variant is through the loss of a functional copy of a gene and reduced gene expression [36]. We, therefore, focused on genes whose 95% confidence interval of absolute fold-change (FC) covers 1.5, corresponding to 50% increase or decrease in gene expression. We reasoned that a degree of de-repression comparable to gaining an extra gene copy would be sufficient to mask the predicted disease mechanism of reduced expression associated with LoF variants [12]. We identified 32 NDD genes that had an absolute FC of ≥ 1.5 and were significantly differentially expressed in at least one of the female groups (group 1, group 2, and group 3) compared to males. Out of these, 17 were X-linked and 15 were autosomal (Additional file 1: Fig. S8). These results indicated that the transcriptional changes induced by de-repression of chromosome X may also influence the penetrance of autosomal variants. ASE information could be obtained for six of the chromosome X genes in the female lines, each displaying higher ASE ratios in the low-XIST lines (Fig. 6A,B), further confirming that the increased expression resulted from erosion of XCI at these genes as a direct compensatory mechanism. These genes demonstrated a dose-dependent increase in expression associated with the higher degree of de-repression. Importantly, however, some of the genes, like *KDM6A* and *TFE3*, displayed considerable line-to-line variation in expression and ASE. This suggested that using *XIST* expression alone may not be sufficient for accounting the variability in XCI in disease modeling.

Given that cell-type-specific pathogenic mechanisms associated with NDD genes are not necessarily present in hiPSCs, we asked whether escape from XCI in the differentiated neurons from the same hiPSCs [37] resulted in compensatory gene expression of these genes. The analysis in the differentiated neurons (16 male, 8 high-XIST female, and 14 low-*XIST* female, Additional file 1: Fig. S9) revealed 16 NDD genes (10 in chromosome X and 6 autosomal) which can reach 50% increase in gene expression in low-*XIST* female neurons (Additional file 1: Fig. S9, Additional file 2: Table S8). Out of the six genes with ASE data available in hiPSCs, three were present in neurons and also displayed increased expression of these genes in the lines with reduced *XIST* levels consistent with de-repression similar to the hiPSCs (Fig. 6C). These results implicated that variation in XCI can directly reduce penetrance of gene disabling variants in hiPSCs and in differentiated cell types, including neurons, and the degree of de-repression of individual genes needs to be considered in biological modeling of X-linked diseases.

## Discussion

Understanding the gene-wise patterns of de-repression and escape from XCI and its influence on sex differences are important for implementing correct study designs for hiPSC-based biological modeling. However, the landscape of XCI has not been systematically surveyed in a large number of lines. Here, we performed a systematic analysis of gene-wise patterns of de-repression and escape from XCI in 165 female hiPSCs using the HipSci resource [14]. The careful analysis of ASE across several female lines expands the previous knowledge of erosion of XCI and provides novel insight into genomic patterns of bi-allelic expression in intact and eroded hiPSCs. Furthermore, by identifying groups of lines with different numbers and degrees of de-repressed genes, and comparison with previous studies of the epigenetic landscape in XCI [15, 25, 26], the data yield mechanistic insight into de-repression and escape from XCI and its impact in regulating genetic sex differences in human cells. With the growing interest for mechanisms of sex differences in the field [38], these data highlight the possibility to use the de-repression patterns of hiPSCs to understand how X chromosome regulates the rest of the genome.

Several previous works have demonstrated global epigenetic variation in hiPSCs that is associated with the irreversible loss of *XIST* expression that leads to the erosion of XCI in some lines [2, 3, 15, 25]. These studies have suggested screening cell lines for markers of XCI, including the loss of *XIST* expression to benchmark the quality of the cells. Here, the analysis of de-repression at the level of individual genes provides a more nuanced view of the erosion of XCI. We found that the fraction of genes with a degree of de-repression increased in lines prior to a considerable decrease in the *XIST* expression in the culture. These high-*XIST* high-ASE lines (group 2) had a similar fraction of de-repressed genes as the lines with lowest *XIST* level (group 3). Similarly, the mean ASE increased sharply in the female lines simultaneously with the fraction of de-repressed genes that was followed by a slower rate of increase in the mean ASE prior to complete loss of *XIST*. Beyond the plateauing changes of ASE of the X chromosome in the eroding lines, the autosomal gene expression differences increased substantially with the complete loss of *XIST* in the cultures. The non-linear relationship between *XIST* levels and de-repression in culture could arise due to a gradually growing number of cells that have lost *XIST*, as suggested previously [16]*.* Alternatively, also supported by the progressive increase of differentially expressed genes in the eroding lines, the loss of *XIST* and de-repression takes place gradually in all cells in a culture and may involve discrete stages and transitions in the XCI erosion [15]. These results underline the importance of applying genome-wide analysis for accurate and sensitive classification of XCI state in the hiPSCs, as suggested previously [4].

In humans, XCI is tightly regulated, and different tissues generally have uniform patterns of escape from XCI [10, 22]. We found that the most intact hiPSCs recapitulated the somatic landscape of escape [10]. The data further revealed that the de-repression associated with erosion of XCI was, similarly, non-random and affected regionally clustered genes along the X chromosome, as noted previously [15]. These included a significantly higher degree of de-repression of genes that escaped in intact hiPSCs and in humans [10]. Furthermore, the reactivated genes were enriched at sites with erosion-associated hypomethylation and remodeling of repressive H3K27me3 domains associated with XCI erosion [15, 25]. Previously, studies in mice suggested that the reactivation of XCI during iPSC reprogramming takes place in a hierarchical order so that genes that are de-repressed early are located near genes that escape XCI [39]. The escape from XCI is controlled by chromatin accessibility in the Xi. *XIST* expression is required for the formation of two densely packed super domains in the Xi with only regions of escaping genes preserving local structure of topologically associated domains with accessibility to the DNA [40]. Our data suggest that similar regional mechanisms that make some genes susceptible for escape from the XCI in human tissues may regulate the de-repression from XCI as well. However, more studies are needed to understand the exact mechanisms that make some genes more likely to escape XCI than others.

One of the key insights revealed by the study is how variability in de-repression affected male–female differences in gene expression. The dose-dependent association between the degree of de-repression and the magnitude in the associated male–female differences in gene expression at autosomes implied that erosion of XCI directly regulated the expression of these genes. This underlines that the de-repression in female hiPSCs—often considered disadvantageous—could yield mechanistic insights into sex effects. We found that de-repression of male-biased expression at autosomes was enriched for genes previously reported to be bound by *XIST* [26], providing mechanistic insights into the roles of the X chromosome and *XIST* in regulating autosomal gene expression. Nevertheless, the variability in de-repression impacted the expression of numerous genes both in the X chromosome and autosomes. Previously X-linked *Dusp9* and *Klhl13* were reported to mediate X dosage effect on autosomal gene expression in mouse [41]. While there was no difference in the expression of human ortholog *DUSP9*, *KLHL13* became de-repressed with XCI erosion, it was female-biased across all the comparisons and its expression appeared to increase with de-repression, suggesting it may contribute to the autosomal sex biases also in human iPSCs. The variability in erosion of XCI has been previously associated with key properties of hiPSCs, such as the differentiation ability [5, 42]. Intriguingly, we found that *BCOR,* a gene subject to inactivation by XCI in humans, where deleterious mutations are associated with increased proliferation and reduced differentiation ability [23], was bi-allelically expressed in female hiPSCs, suggesting that the de-repression could be advantageous for the stem cell properties of the cells. Conversely, we found several dominant disease-linked genes whose expression was changed by de-repression and suggest that these modifying effects can complicate biological modeling if not addressed appropriately. Exciting progress is made to prevent and restore *XIST* expression and for the de-erosion of hiPSCs [16, 43]. However, until such methods are readily available, it remains critical to account for the variability through careful experimental design.

The extreme skew of XCI resulting from the clonality of Xi in the hiPSCs enables detailed ASE analysis of X chromosomal genes using high-coverage bulk-RNA sequencing. However, it is important to note that without single-cell resolution, we cannot address the variability between individual cells in a culture. Therefore, the observed differences in ASE and gene expression may result from quantitative changes that take place in all cells in a culture or larger changes that occur only in a subset of all cells in a culture as discussed above. In addition, although we show extreme variability between hiPSC lines in fraction of genes with bi-allelic expression, we cannot completely rule out a possibility that some ASE estimates are affected by traces of non-clonality in the cultures. We used allelic expression of *XIST*, only expressed from the same Xi in clonal cultures, to verify clonality of the cultures. We observed a handful of lines with a small degree of bi-allelic expression of *XIST*. However, this was not significantly associated with the median ASE and is therefore unlikely to significantly bias the results. Furthermore, the extreme allelic imbalance in *XIST* for all lines is consistent with a single somatic cell origin for the lines. With the current data set, we are unable to distinguish whether the bi-allelic expression of *XIST* results from non-clonality of the line, sequencing error, or other epigenetic variability in the cultures, such as transcriptional activation of *XIST* in the Xa. Importantly, ASE of *XIST* could only be measured for a subset of cells with sufficient *XIST* mRNA levels and heterozygous coding variants in *XIST*. Therefore, we are unable to confirm the clonality of all lines, and the interpretation of the results is grounded on an assumption—strongly supported by the data and previous reports—that lines with low and high levels of *XIST* are similar in terms of clonality of the cultures.

## Conclusions

By conducting a systematic survey of ASE in female hiPSC lines, we have shown that XCI erosion occurs non-randomly affecting epigenetically variable genes, including those that have a degree of escape from XCI in human tissues. We found that the XCI erosion was associated with gene expression differences at sites that undergo epigenetic changes during XCI erosion. In the X chromosome, the erosion led to upregulation of genes at sites that normally carry repressive histone marks and were enriched for regions that undergo hypomethylation during XCI erosion in line with epigenetic modification driving the de-repression in the eroding Xi. In autosomes, the XCI erosion was associated with dose-dependent sex differences, implying direct X-linked mechanisms for regulating autosomal gene expression. We found that the XCI erosion resulted in male-biased expression at several autosomal genes that undergo hypermethylation during the erosion. Furthermore, the loss of *XIST* during XCI erosion increased the expression of genes that are normally bound by *XIST* in the female lines that resulted in the loss of male-biased expression in the eroding lines. Our findings highlight the complex patterns and genomic mechanisms driving the gene expression differences associated with X chromosome and XCI erosion in female hiPSCs.

## Methods

### Statistics

The details about the data sets used in this study and the methods we used in each part of the analyses are elaborated in the following sections. When comparing data from different groups, either *t*-test or Wilcoxon rank-sum test were used according to the normality of the data. The paired versions of the tests were used when comparing paired data coming from the same individual cell lines. The binomial test was used to compare proportions, and Fisher’s exact test or Chi-squared test was used when analyzing contingency tables. Kolmogorov–Smirnov test was used to test the equality of two distributions. A multiple testing correction was applied when necessary. The statistical tests used in each figure and the associated *p*-values have been separately indicated in the corresponding figure legends.

### Data set

The data used in this study have been previously generated by the HipSci project [14] and is available at the HipSci Cell Lines and Data Browser [44]. This study included 282 cell lines (117 male and 165 female) that were derived from the skin tissues of healthy donors with predicted European ancestry, grown in feeder-free culture, and had the open access data available at the HipSci portal for RNA-seq and Exome-seq analyses (Additional file 2: Table S1). The RNA-seq fastq files were used in RNA-seq data processing for DE analysis, variant call files (vcf, whole exome sequencing mpileup variant calls), and RNA-seq bam files (RNA-seq mapped using splice-aware STAR) were used in ASE analysis and can be accessed with study accession numbers PRJEB7388 (for RNA-seq fastq and bam files) and PRJEB7243 (for the whole-exome sequencing mpileup variant calls) in European Nucleotide Archive (ENA). Fastq files from only one sequencing center per cell line were included in the study. The fastq files for sensory neurons differentiated previously from 38 (16 male and 22 female) of the hiPSC lines [37] were downloaded from ENA (PRJEB18630). In addition to the meta-data of hiPSC lines used in this study, information about the differentiated sensory neurons are also listed in Additional file 2: Table S1.

### RNA-seq data processing

RNA-seq data processing was conducted using a pipeline similar to the one used by the GTEx project [45]. More specifically, Homo_sapiens_assembly38.fasta (GRCh38) downloaded from the BROAD Institute GATK resources [46] was used as reference genome, and ALT, HLA, and Decoy contigs were excluded from the reference genome fasta file using the Python code provided in the GTEx RNA-seq pipeline [45]. The comprehensive gene annotation file gencode.v26.annotation.gtf was downloaded from GENCODE [47], and all isoforms of a gene were combined into a single transcript using the collapsed gene model as explained in the GTEx pipeline [45]. Prior to this, salmon package [48] was used to confirm that HipSci lines had been sequenced using an unstranded protocol, similar to the GTEx samples.

The Illumina adapter sequences were trimmed using trimmomatic version 0.39 [49] and the trimmed fasta files were aligned to the reference genome using STAR version 2.6.0c [50]. Gene counts were estimated using RNA-SeQC version 2.3.5 [51]. The gene counts from technical replicates were summed together for each cell line. The same RNA-seq data processing pipeline was used for the hiPSC lines and the differentiated sensory neurons.

### Differential gene expression analysis

Differential expression (DE) analysis was conducted on normalized gene counts for protein-coding genes and long intergenic noncoding RNAs (16,831 and 15,369 genes in total for hiPSCS and differentiated sensory neurons, respectively). For hiPSCs, we performed two separate DE analyses: one in which females were divided into two groups (low *XIST* and high *XIST*) and one in which females were divided into three groups according to *XIST* expression and fraction of bi-allelic genes (groups 1, 2, 3). For differentiated sensory neurons, the DE analysis was performed for females with low *XIST* and high *XIST*. In each analysis, the genes that had less than ten counts were filtered out. A TMM (trimmed mean of M-values) normalization was used for the gene-wise read counts and converted to log_2_(CPM + 0.5) in the limma-voom package [52]. In addition, surrogate variables, which were adjusted for in the DE analyses, were estimated by the sva R package [53]. For the DE analysis, a mixed linear framework, implemented in the DREAM method [54], was used that allowed accounting for the correlation between multiple replicate samples obtained from the same donor. The DE analysis for the sensory neurons included only one replicate sample per each donor and limma-voom [52] was used for the DE analysis with the edgeR R package [55–57]. The DE analyses for hiPSCs included the following comparisons: high-XIST females vs. low-XIST females, males vs. group 1 females, males vs. group 2 females, males vs. group 3 females, group 1 females vs. group 2 females, and group 2 females vs. group 3 females (Additional file 2: Table S6), and for differentiated sensory neurons: males vs. high-XIST females and males vs. low-XIST females (Additional file 2: Table S8). Genes with adjusted *p*-value < 0.05 were considered as differentially expressed in each comparison. All statistical analyses were performed using R Statistical Software (v4.0.5) [58]. Figures were produced by using ggplot2 [59] and ComplexUpset [60, 61] libraries.

### Allele-specific expression (ASE) analyses

To estimate the read counts which mapped to bi-allelic variants in each hiPSC line, the Genome Analysis Toolkit GATK [58] was used with the HipSci vcf (exome-seq mpileup) and RNA-seq bam files. For each line, GATK (version 4.2.0.0) ASEReadCounter was run with options -min-mapping-quality 10 and –min-base-quality 2, using the hs37d5 [59] assembly and GencodeV19 annotation [59] of the human reference genome. All bi-allelic variants that had at least 20 reads mapped to them were included in further ASE analysis. When calculating the gene-level ASE, among all the variants located within a gene, only the variants which possessed the bigger count size if there was a gap less than the read length (100 bp) between any two variants were considered. The alleles which had the smaller number of counts (minor alleles), with respect to their counterparts, were assumed to be located on the same chromosome haplotype. ASE was calculated for each gene by dividing the total number of reads that mapped to the minor alleles to the total number of reads that mapped to all alleles. Only the bi-allelic variants located along the gene were included in the analysis. ASE could be calculated for 12,500 autosomal and 411 X chromosome genes in at least one of the hiPSC lines. For each X chromosome gene per line, an alternative hypothesis of ASE > 0.1 was tested using a one-sided binomial test to determine whether the gene was bi-allelically expressed (FDR *q*-value < 0.01). The ASE > 0.1 threshold was selected based on previous literature to facilitate comparison, particularly with human tissues. Furthermore, the selected threshold was reasoned to be robust for defining ASE in cases with small degree of bi-allelic expression of XIST (ASE: 0.05–0.1) to avoid bias from the small fraction of chimeric cell populations in the studied cell lines. The clonality of cell lines was tested by introducing a stricter ASE threshold (ASE < 0.05) for the *XIST* gene. *XIST* was defined to be monoallelically expressed if the null hypothesis of ASE ≥ 0.05 was rejected for the *XIST* gene by the one-sided binomial test. It is important to note that the RNA-seq data was not realigned with a SNP-aware method, which may cause a bias toward the reference allele in the ASE results. To rule out a reference allele bias in diluting the ASE estimates in group 1, the proportion of reads mapping to the reference allele was calculated. Group 3 had a slightly higher proportion of reference allele than the rest. The stronger bias in group 3 suggested that the true ASE is slightly higher in this group than the estimated ASE. The bias was observed primarily for genes with a clear bi-allelic expression with ASE ranging 0.3–0.5 (Fig. S5E).

### Grouping of female lines by escape from XCI

A threshold for normative *XIST* expression was defined from *XIST* expression in human tissues from GTEx_Analysis_v8 results available at the GTEx portal [62]. A threshold for loss of *XIST* was defined as having lower *XIST* mRNA level than observed for any female sample in any tissue in GTEx (< 1.5 log_2_CPM). The *XIST* expression threshold of 1.5 log_2_CPM was used to divide the female hiPSCs as well as the female differentiated sensory neurons into two groups as low *XIST* and high *XIST* females. By combining ASE information with *XIST* expression level, a more refined grouping of female hiPSCs was obtained. For this, a k-means clustering (*k* = 3) was performed using the standardized fraction of genes with ASE > 0.1 (one-sided binomial test, FDR *q*-value < 0.01) and the standardized *XIST* expression levels. The gap statistic method [24] suggested an optimal number of three clusters named group 1, group 2, and group 3 females.

### GO term enrichment analysis

Using the clusterProfiler R package [63], a GO term enrichment analysis was performed for the gene groups which were differentially expressed between males and different female groups. The enrichGO function in the clusterProfiler R package was used with default settings for the parameters and *p*valueCutoff of 0.05 and *q*valueCutoff of 0.10. More specifically, we focused on the genes which were downregulated or upregulated only in group 3 females (*N* = 2729), only in group 2 females (*N* = 321), and the genes which had shared effects in group 2 and group 3 females (*N* = 2018). The results can be found in Additional file 2: Table S7.

### XIST-binding sites enrichment analysis in autosomes

In this analysis, all conserved autosomal *XIST* peaks of human naïve-hPSCs were included (Additional file 2: Table S4 from [26]). The overlap between the genes annotated as having *XIST* peaks and the genes significantly DE in each sex-DE analyses (male vs female group 1, male vs female group 2, and male vs female group 3) was computed. The DE gene lists by direction of effect (male-/female-biased) were separated. Then over-representation and depletion were tested using a hypergeometric test. The relative enrichment effect size was computed as follows: (number of overlapping genes / number of XIST peaks) / (number of significant DE genes / number of analyzed genes). Finally, the same statistical tests and relative enrichment for 1000 random gene sets was tested to compare how extreme our enrichment was.

### Methylation enrichment analysis

In this analysis, methylation data from human female iPSC/ESCs going through XCI erosion [25] were used. The methylation change (hypo- or hyper-methylation) of probes at the genes’ promoter (transcription start site (TSS) ± 1500 bp) both on autosomes and the X chromosome separately was used. Bansal et al. classified the methylation change in five different transitions along XCI erosion trajectory, with transition 1 being the earliest transition and transition 5 the latest [25]. For all transitions separated by the direction of methylation change (hypo- or hyper- methylation), the overlap was computed with the genes significantly DE in each sex-DE analyses (male vs female group 1, male vs female group 2, and male vs female group 3). Once again, we separated the DE gene lists by direction of effect (male-/female-biased). Then, over-representation and depletion were tested for using a hypergeometric test and the relative enrichment effect size was computed. And finally, the same test and metric for 1000 random gene sets were used.

### H3K27me3 and H3K9me3 enrichment analysis in X-chromosome

In this analysis, H3K27me3 and H3K9me3 histone marks on the X-chromosome of H9 cell line with a normal (XaXi, one active and one inactive X-chromosome) or eroded (XaXe, one active and one “eroded” X-chromosome) XCI status [15] were used. The histone mark peaks were assigned to a gene if they overlapped the promoter (TSS ± 3000 bp) or the gene body (UTRs, exons or introns) using the ChIPseeker R package [64]. For each histone mark in the two cell lines, the overlap with the genes that are significant in each DE analysis between female groups (group 1 vs group 2, group 1 vs group 3, and group 3 vs group 2) was computed. Once again, the DE gene lists were separated by direction of effect. Then, over-representation and depletion were tested for using a hypergeometric test and the relative enrichment effect size was computed. Finally, the same test and metric were computed for 1000 random gene sets.

### Supplementary Information

Additional file 1: Supplementary Fig. S1 – Fig. S9 and Supplementary figure legends.

Additional file 2: Supplementary tables. Table S1 – Table S8.

Additional file 3: Review_history.

## Data Availability

All raw data used in this article were previously generated by HipSci project and accessible through the HipSci cell lines and data browser at [65]. RNA-seq data for hiPSCs and differentiated sensory neurons are available in European Nucleotide Archive (ENA) with accession numbers PRJEB7388 [66] and PRJEB18630 [67], respectively. Whole-exome sequencing mpileup variant data for iPSCs are available in ENA with accession number PRJEB7243 [68]. The processed data supporting the conclusions of this article are included in the supplementary tables. The codes and data used in this study are available at [69, 70] under an MIT license.
