# Supplementary material for: X-chromosome inactivation in human iPSCs provides insight into X-regulated gene expression in autosomes
Source: Genome Biol. 2024 May 31;25:144. doi: 10.1186/s13059-024-03286-8 (PMC11143737; doi:10.1186/s13059-024-03286-8)
Supplement: Supplementary file 1 — Additional file 1: Supplementary Fig. S1 – Fig. S9 and Supplementary figure legends. [file 13059_2024_3286_MOESM1_ESM.pdf]

Supplementary figures

Fig. S1. *XIST* expression in female human tissues in GTEx and hiPSCs. All female GTEx tissues had an *XIST* expression level of at least 1.5 $\log_2$CPM (indicated by the dashed horizontal line). Female hiPSCs displayed a wider range of *XIST* expression ranging below 1.5 $\log_2$CPM that was used to define a threshold for low *XIST.*

Fig. S2. ASE of X chromosome genes in high and low XIST female lines. A) Median and B) mean ASE of X chromosome genes in female hiPSC lines with high or low level of *XIST* (n=118 and 47, respectively). Female lines with low levels of XIST had significantly larger median ASE (p-val = 4.6e-03) and mean ASE (p-val = 3.4e-11) than those with high levels of XIST (one-sided Wilcoxon rank-sum test). C) Fraction of genes with ASE > 0.1 does not differ between lines with monoallelic expression of *XIST* (ASE < 0.05, one-sided binomial test, p-value < 0.05) and lines with traces of bi-allelic expression (ASE ≥ 0.05) of *XIST* (p-value = 0.27, Wilcoxon rank-sum test). D) A boxplot of fraction of cell lines where gene ASE > 0.1 for genes that are known to at least variably escape in humans and that are inactive in humans in hiPSC lines with high or low level of *XIST* mRNA. Only the genes which had ASE data in at least 10 cell lines in both low-XIST and high-XIST female lines are included, excluding *XIST* and the PAR genes. The fraction of cell lines with ASE > 0.1 is significantly higher in the female lines with low *XIST* levels than in the female lines with high *XIST* levels for the genes which are known to escape or variably escape in humans (paired one-sided Wilcoxon rank-sum test, p = 1.49e-08) as well as for the genes which are known to be inactive in humans (paired one-sided Wilcoxon rank-sum test, p = 4.64e-13). E) A cumulative distribution plot of the percentage of genes in group 1, group 2, and group 3 female hiPSCs based on the fraction of cell lines where the genes show bi-allelic expression (ASE > 0.1, one-sided binomial test, FDR q-value < 0.01). All female hiPSC lines with ASE data available are included.

Fig. S3. Bi-allelic expression for 139 X chromosome genes in 165 female lines. The heatmap and bar graphs summarize the bi-allelic genes (ASE > 0.1, one-sided binomial test, FDR q-value < 0.01) in hiPSCs. The bar graphs above heatmap summarize (i) the average number of sequence reads per gene at an ASE site (ii) and number of genes with available ASE data for each line. The number of informative genes is not significantly associated with the *XIST* levels

(p-value = 0.30, Spearman's rank correlation test). We observed a slight negative relationship between the average number of reads per gene at an ASE site and the *XIST* levels (p-value = 0.003, Spearman's rank correlation coefficient = -0.23). This is expected given that loss of *XIST* is associated with higher gene expression from the X chromosome. iii) The heatmap displays the 139 genes (rows) that are bi-allellic (red) or monoallelic (blue) for each cell line (columns). Cells in gray color indicate missing information. The low- and high-XIST lines are separated by a vertical line. The genes are ordered by location in X the chromosome and the lines are In decreasing order by *XIST* level. Names of the genes with significantly (p-value < 0.05, Fisher's exact test) higher fraction of cell lines with bi-allelic expression in low *XIST* females than in high *XIST* females are colored in red. The bar graphs on the right side indicate (iv) the number of lines with available ASE data for each gene, and (v) the average number of reads per gene at an ASE site across the lines vi) A bar graph displaying the difference in the fraction of lines in which the genes show bi-allelic expression between low and high *XIST* females. The nominally significant differences for 41 genes (p-value < 0.05, Fisher's exact test) are indicated in black.

Fig. S4. Standardized gene expression for X chromosome genes matched with ENSEMBL identifiers (GencodeV19) with Figure 2B and Figure S4 genes (excluding ENSG00000250349 / TM4SF2 with no data) in 165 female lines (columns). The cell lines are ordered in decreasing order of *XIST* expression. The low and high *XIST* lines are separated by a vertical line. The genes (rows) are ordered by location in the X chromosome from top (p-arm) to bottom (q-arm). The bar graph on the right-side displays differences in gene expression (log_2(fold change)) between the low and high *XIST* lines and the significant differences (adjusted p-value < 0.05) are highlighted in black for 71 genes. The log2(fold change) for XIST is cropped at -0.5, which was originally -6.51. Names of the genes with significant differences in their expression between low *XIST* and high *XIST* lines are colored in red.

Fig. S5. De-repression associated gene expression in chromosome X. A) Correlation of mean ASE and fraction of genes with ASE>0.1 in female lines. B) Sigmoid fit of XIST expression level and mean ASE of the X chromosome. The inflection point is highlighted with a vertical line at 6.6 log_2CPM. The line plateaus at mean ASE of 0.2. C) Fraction of genes with ASE > 0.1 is significantly different between group 1 females and group 2 and group 3 females, with the

highest fraction of escaping genes in group 3 female lines (group 1 – group 2: p-value = 1.42×10^{-19}; group 2 – group 3: p-value = 0.32, group 1 – group 3: p-value = 7.93×10^{-20}; Wilcoxon rank-sum test). D) Variation of *XIST* expression within each female group is not associated with mean ASE of the X chromosome. E) A boxplot of the proportion of reads mapped to the reference allele. Group 3 has a significantly higher proportion of sequence reads for the reference allele than group 1 and 2 (p-values 3.6×10^{-6} and 3.2×10^{-3}, respectively; t-test). The reference allele bias may cause reduced ASE estimates. F) The two first principal components (PC1 and PC2) for PCA of autosomal gene expression. The cell lines are colored by sex and female XCI group (male: blue, females in group 1: red, group 2: violet, group 3: pink) G) Full sized upset plot for differentially expressed X chromosome genes (see Figure 4A).

Fig. S6. Genomic mechanisms of de-repression of XCI. A,B) A relative enrichment of differentially expressed genes between the female groups: group 1-group2 (G1G2), group 1-group3 (G1G3), and group 3-group2 (G3G2) at regions of repressive heterochromatin marks for H3K27Me3 and H3K9Me3 in eroded (XaXe) and inactive (XaXi) cell lines. Genes that are upregulated in group 2 and group 3 compared to group 1 are enriched for regions with H3K27Me3 (A) in inactive cells (adjusted p-value < 0.05, denoted with asterisk). Overlap with 1000 random gene sets are presented in violin plots. No enrichment was detected for the eroded X chromosome XaXe for H3K9Me3 (B). C) A schematic presentation of progressive methylation changes associated with XCI erosion transitions 1–5, modified from (25). D) A relative enrichment of female- and male- biased genes (red, blue, respectively) in group 2 for X chromosome hypomethylation associated with progressive transitions of XCI erosion. Female-biased genes in group 2 are significantly enriched for hypomethylation associated with transition 1 of XCI erosion (denoted with asterisk). Overlap with 1000 random gene sets is shown as violin plot. E) The percentage of differentially expressed (DE) X-linked genes with female (upper) or male (lower) biased expression and hypomethylated promoter in the X chromosome at XCI transitions 1–5. F-G) The percentage of autosomal DE genes with female (upper) or male (lower) biased expression and hypomethylated (F) or hypermethylated (G) promoter at XCI transitions 1–5.

Fig. S7. De-repression associates with changes in autosomal gene expression. A) The full-sized upset plot described in Figure 5A. B) A scatter plot for log fold changes in group 1 (x-axis) and

group 3 (y-axis) females for 2,018 genes with significant sex-effects in group 2 and group 3. A diagonal dashed line in black. The genes are colored by the group with larger effect (pink in group 3 and red in group 1). C) Boxplots of the differentiation efficiency for iPSC-derived endoderm, data taken from (23) for the same cell lines in male and group 1, group 2 and group 3 females. Wilcoxon rank-sum test p-values are shown between different groups, suggesting slightly lower differentiation ability for group 2 and group 3 female lines than the group 1 female lines. D) Log-fold changes of male-biased genes that are bound or unbound by *XIST*. E) Enrichment of hypermethylated promoters associated with XCI erosion trajectory (25) in autosomal sex-DE genes: male vs female group 1 (at the top) and male vs female group 2 (at the bottom). Same representation as in Figure 4D and 4E. Asterisk indicates a significant over-representation or depletion with a hypergeometric test (p-value < 0.05/36 ~ 0.001). F) Enrichment of hypomethylated promoters associated with XCI erosion trajectory(25) in autosomal sex-DE genes: male vs female group 1 (at the top), male vs female group 2 (in the middle) and male vs female group 3 (at the bottom). Same representation as in Figure 4D and 4E. Asterisk indicates a significant over-representation or depletion with a hypergeometric test (p-value < 0.05/36 ~ 0.001).

Fig. S8. Expression of NDD genes in hiPSCs. A) Expression of autosomal genes in the three groups of female lines and male lines. B) Expression of X chromosome genes in the three groups of female lines and male lines.

Fig. S9 NDD gene expression in hiPSC-derived sensory neurons for A) X-chromosome and B) autosomal genes. C) *XIST* expression in differentiated neurons and corresponding iPSCs. *XIST* expression in hiPSCs and differentiated sensory neurons show high similarity with exception of one line (circled).

Fig. S1

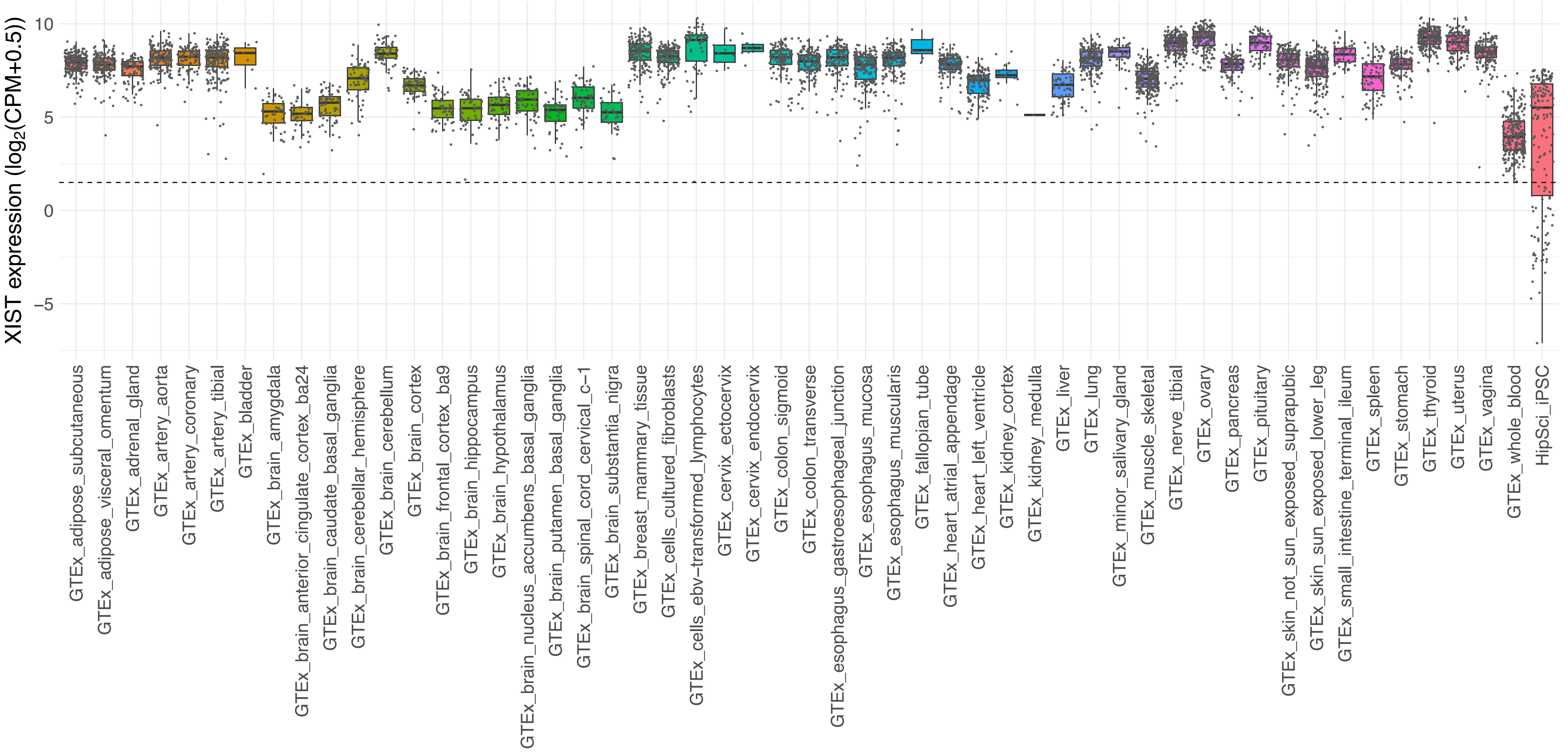
XIST expression (log_2(CPM+0.5))
10
5
0
−5
GTEx_adipose_subcutaneous
GTEx_adipose_visceral_omentum
GTEx_adrenal_gland
GTEx_artery_aorta
GTEx_artery_coronary
GTEx_artery_tibial
GTEx_bladder
GTEx_brain_amygdala
GTEx_brain_anterior_cingulate_cortex_ba24
GTEx_brain_caudate_basal_ganglia
GTEx_brain_cerebellar_hemisphere
GTEx_brain_cerebellum
GTEx_brain_cortex
GTEx_brain_frontal_cortex_ba9
GTEx_brain_hippocampus
GTEx_brain_hypothalamus
GTEx_brain_nucleus_accumbens_basal_ganglia
GTEx_brain_putamen_basal_ganglia
GTEx_brain_spinal_cord_cervical_c−1
GTEx_brain_substantia_nigra
GTEx_breast_mammary_tissue
GTEx_cells_cultured_fibroblasts
GTEx_cells_ebv−transformed_lymphocytes
GTEx_cervix_ectocervix
GTEx_cervix_endocervix
GTEx_colon_sigmoid
GTEx_colon_transverse
GTEx_esophagus_gastroesophageal_junction
GTEx_esophagus_mucosa
GTEx_esophagus_muscularis
GTEx_fallopian_tube
GTEx_heart_atrial_appendage
GTEx_heart_left_ventricle
GTEx_kidney_cortex
GTEx_kidney_medulla
GTEx_liver
GTEx_lung
GTEx_minor_salivary_gland
GTEx_muscle_skeletal
GTEx_nerve_tibial
GTEx_ovary
GTEx_pancreas
GTEx_pituitary
GTEx_skin_not_sun_exposed_suprapubic
GTEx_skin_sun_exposed_lower_leg
GTEx_small_intestine_terminal_ileum
GTEx_spleen
GTEx_stomach
GTEx_thyroid
GTEx_uterus
GTEx_vagina
GTEx_whole_blood
HipSci_iPSC

Fig. S2

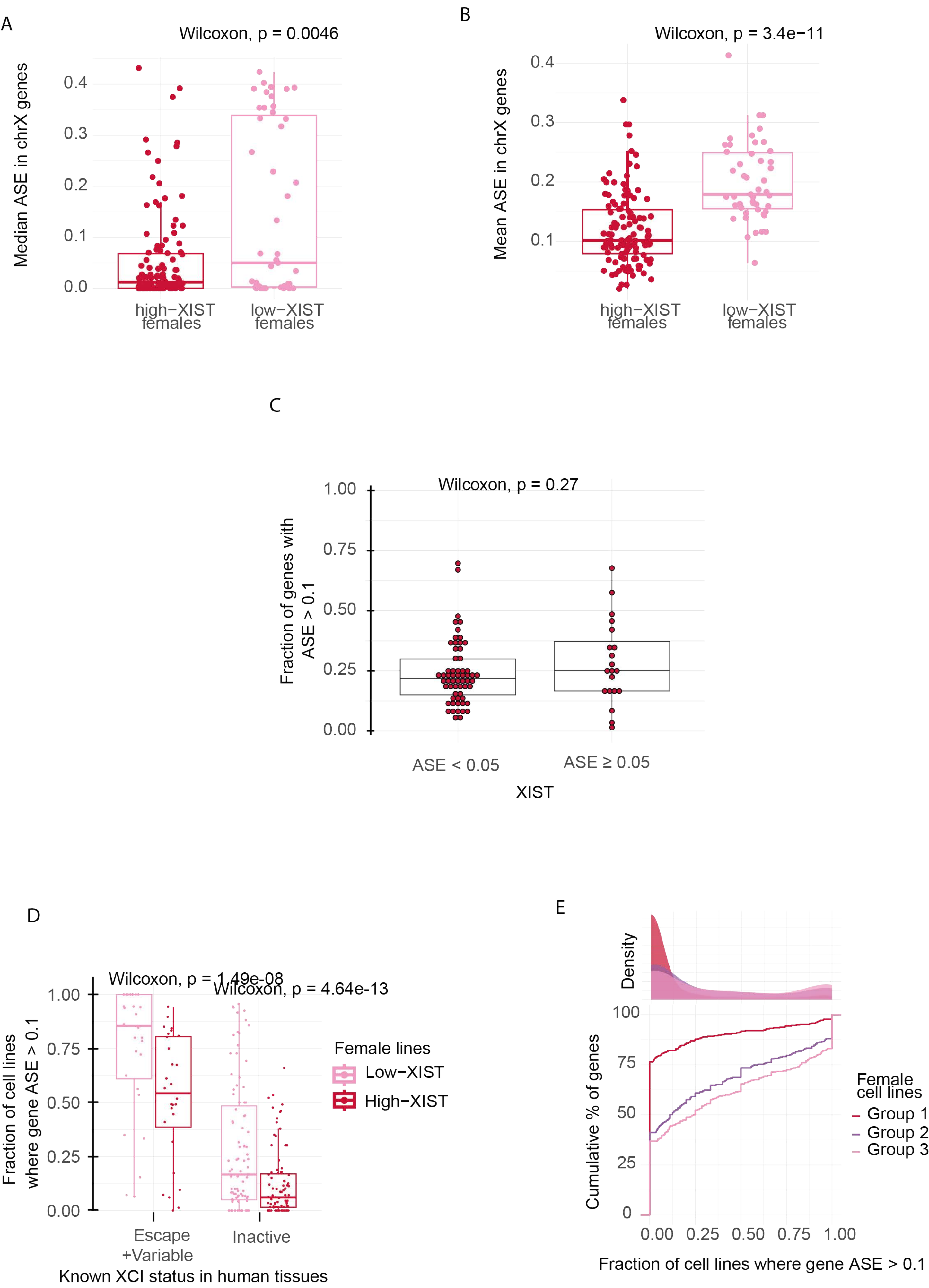

A
Wilcoxon, p = 0.0046
Median ASE in chrX genes
0.4
0.3
0.2
0.1
0.0
high−XIST females
low−XIST females
B
Wilcoxon, p = 3.4e−11
Mean ASE in chrX genes
0.4
0.3
0.2
0.1
high−XIST females
low−XIST females
C
Wilcoxon, p = 0.27
Fraction of genes with ASE > 0.1
1.00
0.75
0.50
0.25
0.00
ASE < 0.05
ASE ≥ 0.05
XIST
D
Wilcoxon, p = 1.49e-08
Wilcoxon, p = 4.64e-13
Fraction of cell lines where gene ASE > 0.1
1.00
0.75
0.50
0.25
0.00
Escape +Variable
Inactive
Known XCI status in human tissues
Female lines
Low−XIST
High−XIST
E
Density
Cumulative % of genes
100
75
50
25
0
0.00
0.25
0.50
0.75
1.00
Fraction of cell lines where gene ASE > 0.1
Female cell lines
Group 1
Group 2
Group 3

Fig. S3

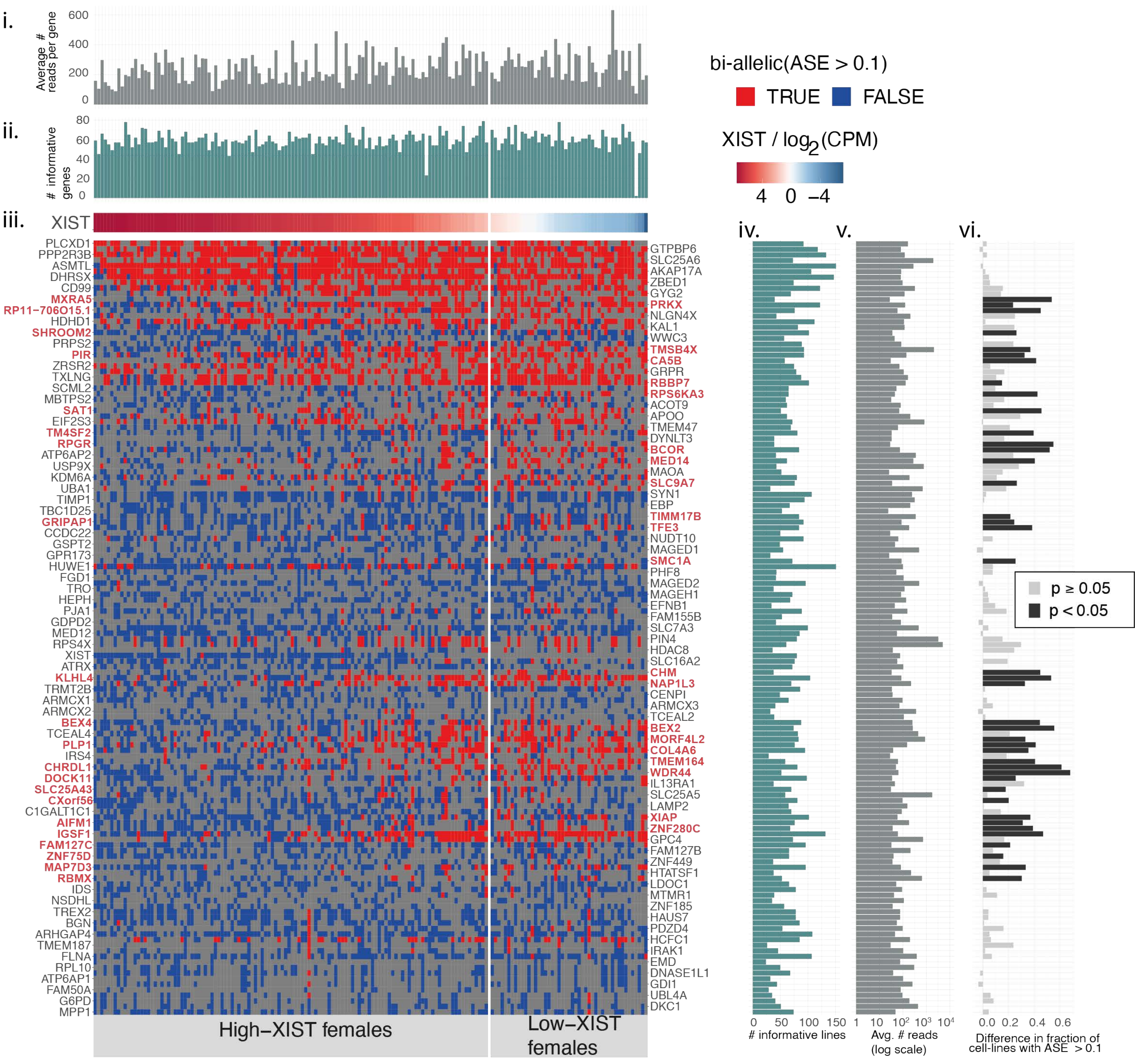

i.
Average # reads per gene
600
400
200
0
ii.
informative genes
80
60
40
20
0
iii.
XIST
bi-allelic(ASE > 0.1)
TRUE
FALSE
XIST / log2(CPM)
4
0
−4
PLCXD1
PPP2R3B
ASMTL
DHRSX
CD99
MXRA5
RP11−706O15.1
HDHD1
SHROOM2
PRPS2
PIR
ZRSR2
TXLNG
SCML2
MBTPS2
SAT1
EIF2S3
TM4SF2
RPGR
ATP6AP2
USP9X
KDM6A
UBA1
TIMP1
TBC1D25
GRIPAP1
CCDC22
GSPT2
GPR173
HUWE1
FGD1
TRO
HEPH
PJA1
GDPD2
MED12
RPS4X
XIST
ATRX
KLHL4
TRMT2B
ARMCX1
ARMCX2
BEX4
TCEAL4
PLP1
IRS4
CHRDL1
DOCK11
SLC25A43
CXorf56
C1GALT1C1
AIFM1
IGSF1
FAM127C
ZNF75D
MAP7D3
RBMX
IDS
NSDHL
TREX2
BGN
ARHGAP4
TMEM187
FLNA
RPL10
ATP6AP1
FAM50A
G6PD
MPP1
GTPBP6
SLC25A6
AKAP17A
ZBED1
GYG2
PRKX
NLGN4X
KAL1
WWC3
TMSB4X
CA5B
GRPR
RBBP7
RPS6KA3
ACOT9
APOO
TMEM47
DYNLT3
BCOR
MED14
MAOA
SLC9A7
SYN1
EBP
TIMM17B
TFE3
NUDT10
MAGED1
SMC1A
PHF8
MAGED2
MAGEH1
EFNB1
FAM155B
SLC7A3
PIN4
HDAC8
SLC16A2
CHM
NAP1L3
CENPI
ARMCX3
TCEAL2
BEX2
MORF4L2
COL4A6
TMEM164
WDR44
IL13RA1
SLC25A5
LAMP2
XIAP
ZNF280C
GPC4
FAM127B
ZNF449
HTATSF1
LDOC1
MTMR1
ZNF185
HAUS7
PDZD4
HCFC1
IRAK1
EMD
DNASE1L1
GDI1
UBL4A
DKC1
High−XIST females
Low−XIST females
iv.
0 50 100 150
informative lines
v.
1 10 10² 10³ 10⁴
Avg. # reads (log scale)
vi.
0.0 0.2 0.4 0.6
Difference in fraction of cell-lines with ASE > 0.1
p ≥ 0.05
p < 0.05

Fig. S4

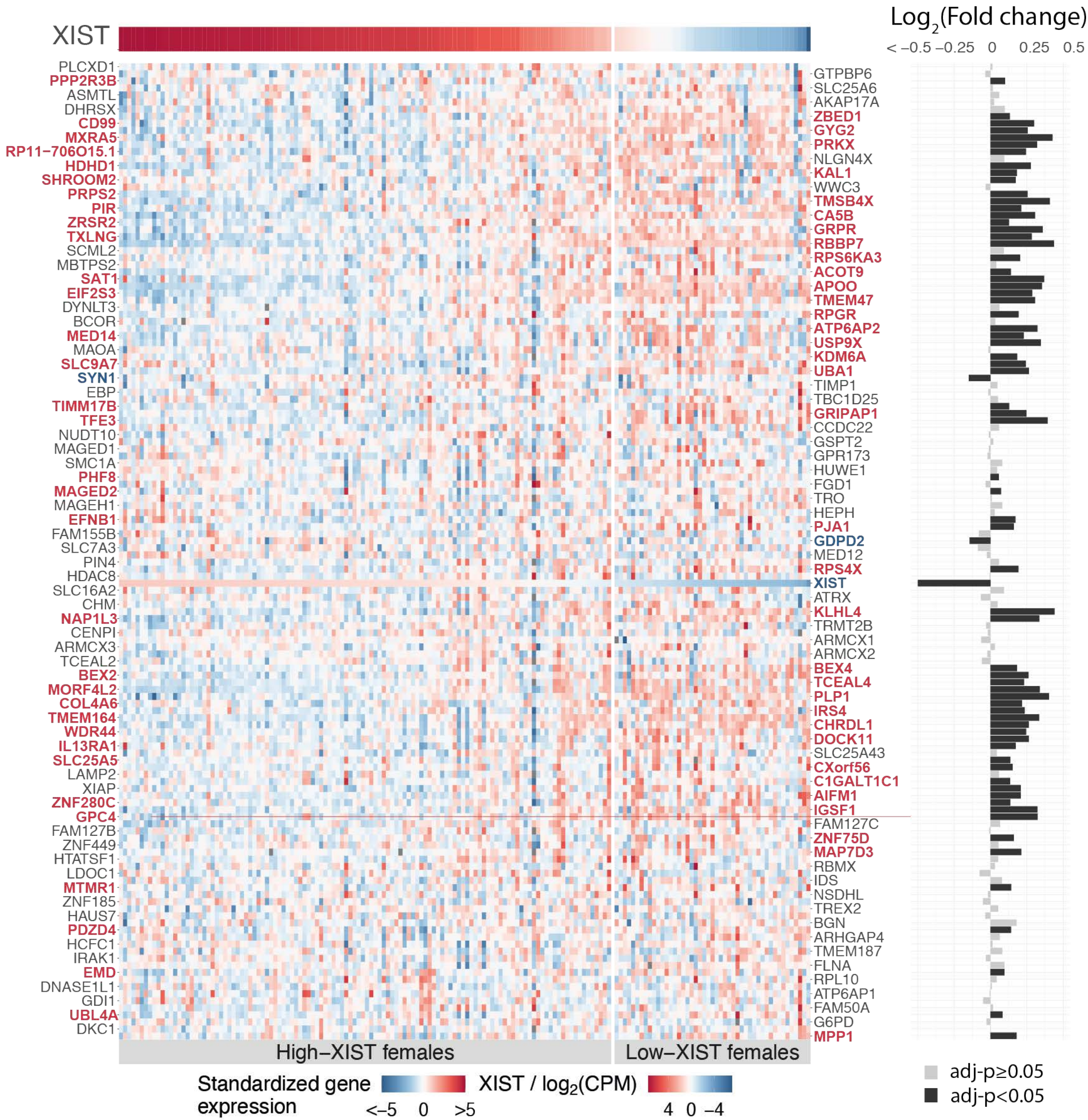

XIST
Log2(Fold change)
< −0.5 −0.25 0 0.25 0.5
PLCXD1
PPP2R3B
ASMTL
DHRSX
CD99
MXRA5
RP11−706O15.1
HDHD1
SHROOM2
PRPS2
PIR
ZRSR2
TXLNG
SCML2
MBTPS2
SAT1
EIF2S3
DYNLT3
BCOR
MED14
MAOA
SLC9A7
SYN1
EBP
TIMM17B
TFE3
NUDT10
MAGED1
SMC1A
PHF8
MAGED2
MAGEH1
EFNB1
FAM155B
SLC7A3
PIN4
HDAC8
SLC16A2
CHM
NAP1L3
CENPI
ARMCX3
TCEAL2
BEX2
MORF4L2
COL4A6
TMEM164
WDR44
IL13RA1
SLC25A5
LAMP2
XIAP
ZNF280C
GPC4
FAM127B
ZNF449
HTATSF1
LDOC1
MTMR1
ZNF185
HAUS7
PDZD4
HCFC1
IRAK1
EMD
DNASE1L1
GDI1
UBL4A
DKC1
GTPBP6
SLC25A6
AKAP17A
ZBED1
GYG2
PRKX
NLGN4X
KAL1
WWC3
TMSB4X
CA5B
GRPR
RBBP7
RPS6KA3
ACOT9
APOO
TMEM47
RPGR
ATP6AP2
USP9X
KDM6A
UBA1
TIMP1
TBC1D25
GRIPAP1
CCDC22
GSPT2
GPR173
HUWE1
FGD1
TRO
HEPH
PJA1
GDPD2
MED12
RPS4X
XIST
ATRX
KLHL4
TRMT2B
ARMCX1
ARMCX2
BEX4
TCEAL4
PLP1
IRS4
CHRDL1
DOCK11
SLC25A43
CXorf56
C1GALT1C1
AIFM1
IGSF1
FAM127C
ZNF75D
MAP7D3
RBMX
IDS
NSDHL
TREX2
BGN
ARHGAP4
TMEM187
FLNA
RPL10
ATP6AP1
FAM50A
G6PD
MPP1
High−XIST females
Low−XIST females
Standardized gene expression
<−5 0 >5
XIST / log2(CPM)
4 0 −4
adj-p≥0.05
adj-p<0.05

Fig. S5

Place holder for

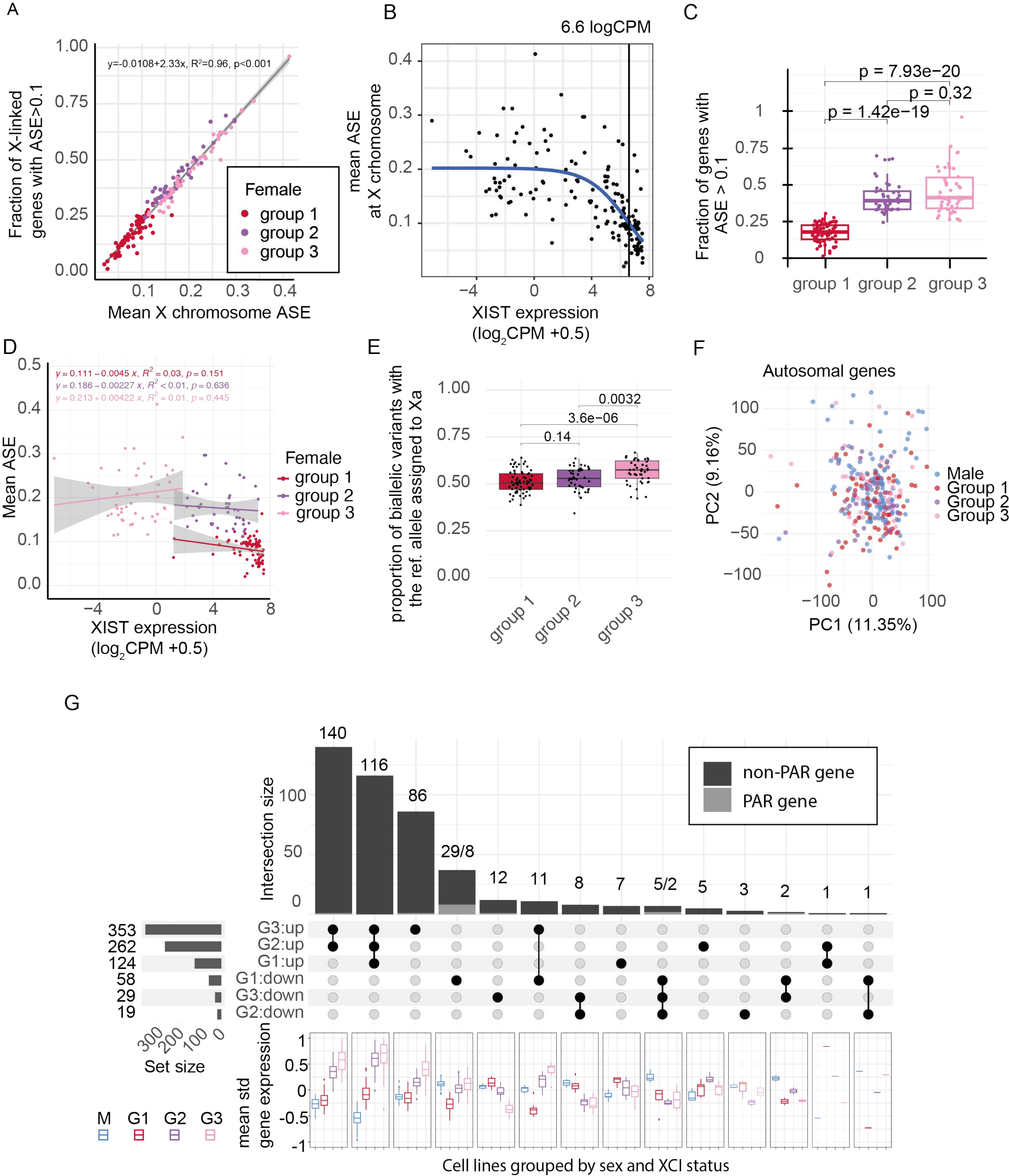

Fig. S6

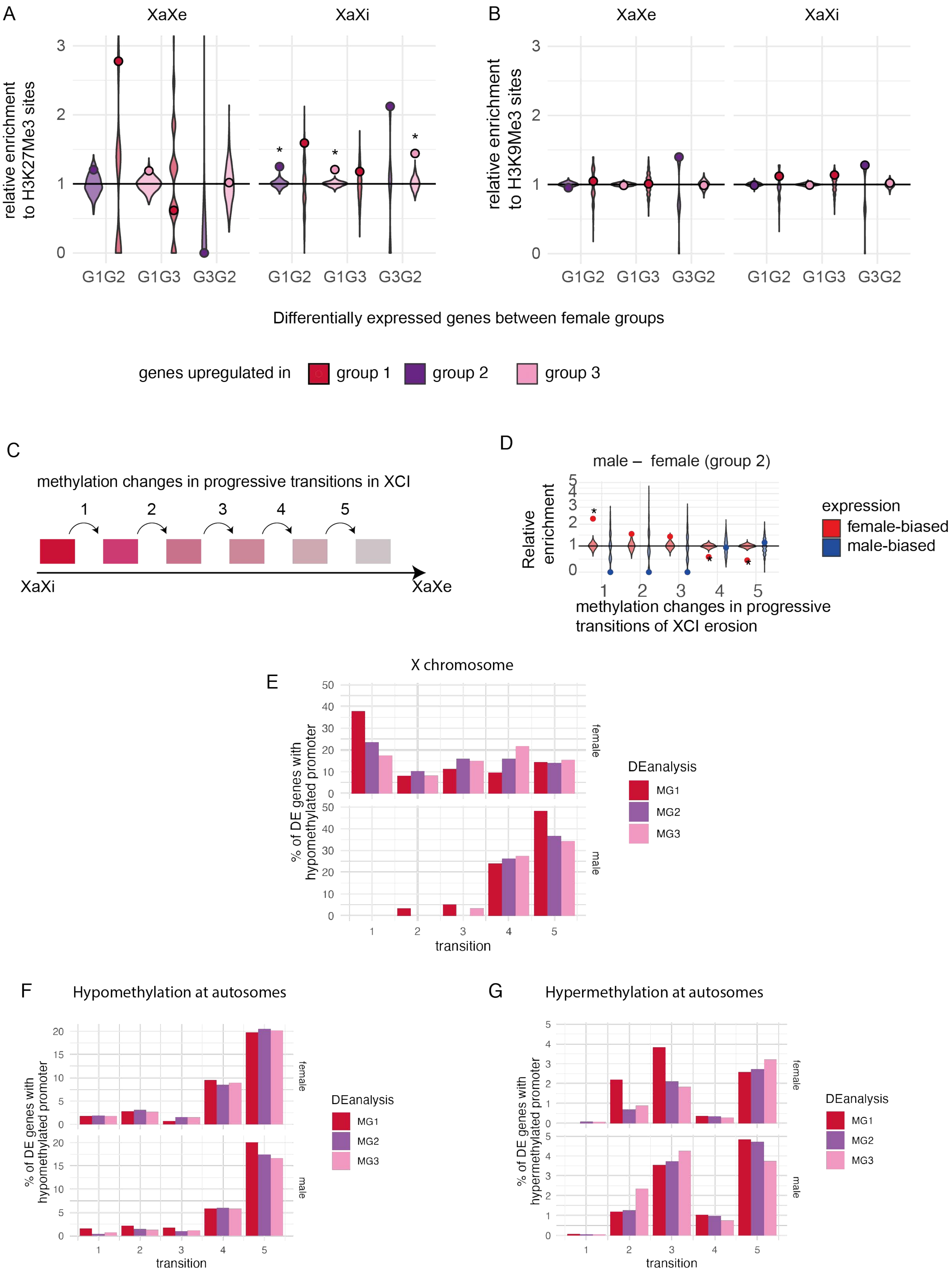
A
XaXe
XaXi
relative enrichment to H3K27Me3 sites
3
2
1
0
G1G2 G1G3 G3G2
G1G2 G1G3 G3G2
*
*
*
B
XaXe
XaXi
relative enrichment to H3K9Me3 sites
3
2
1
0
G1G2 G1G3 G3G2
G1G2 G1G3 G3G2
Differentially expressed genes between female groups
genes upregulated in
group 1
group 2
group 3
C
methylation changes in progressive transitions in XCI
1 2 3 4 5
XaXi
XaXe
D
male – female (group 2)
Relative enrichment
5 4 3 2 1 0
*
*
*
1 2 3 4 5
methylation changes in progressive transitions of XCI erosion
expression
female-biased
male-biased
E
X chromosome
% of DE genes with hypomethylated promoter
50 40 30 20 10 0
female
male
1 2 3 4 5
transition
DEanalysis
MG1
MG2
MG3
F
Hypomethylation at autosomes
% of DE genes with hypomethylated promoter
20 15 10 5 0
female
male
1 2 3 4 5
transition
DEanalysis
MG1
MG2
MG3
G
Hypermethylation at autosomes
% of DE genes with hypermethylated promoter
5 4 3 2 1 0
female
male
1 2 3 4 5
transition
DEanalysis
MG1
MG2
MG3

Fig. S7

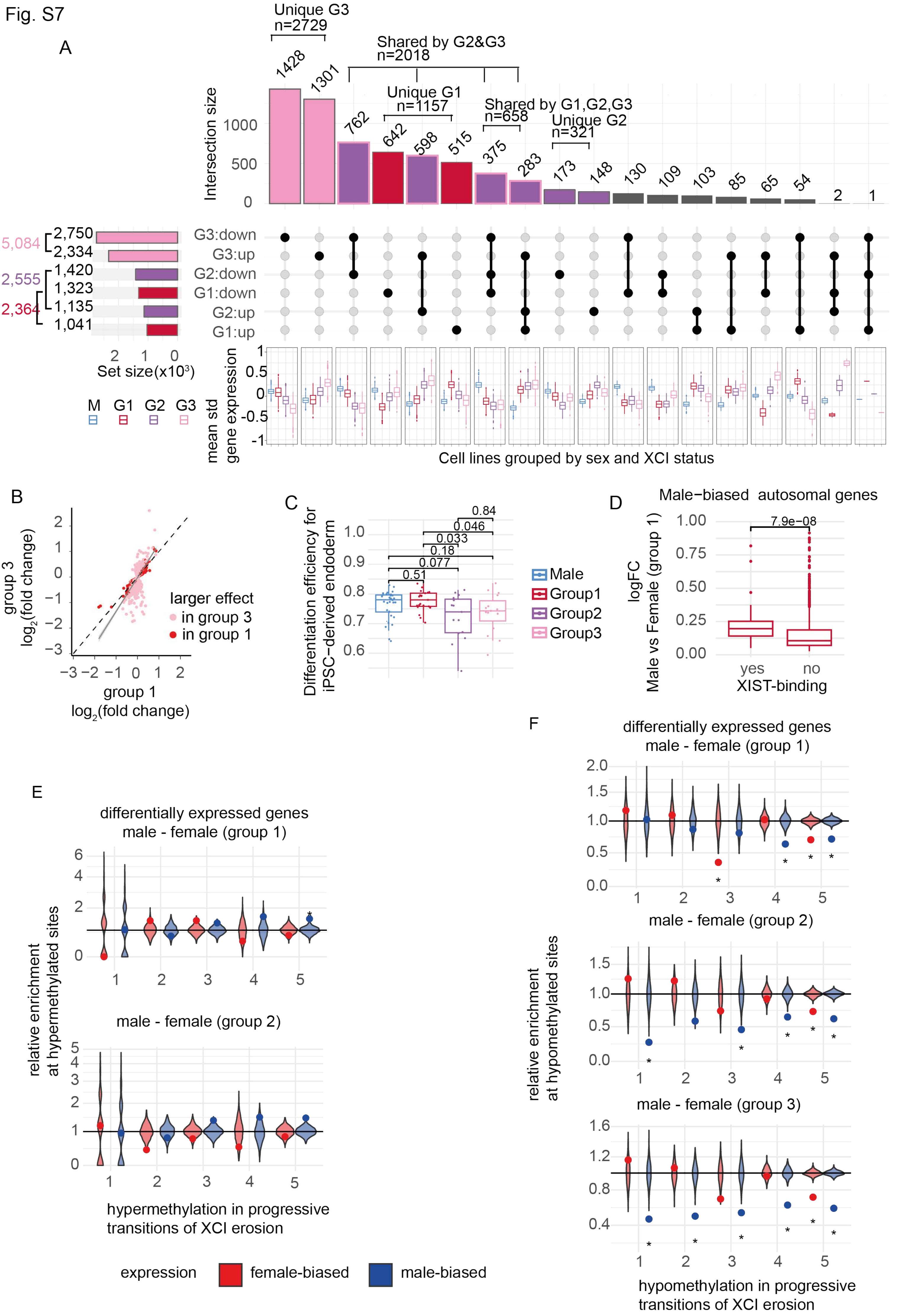

A
Unique G3
n=2729
Shared by G2&G3
n=2018
Unique G1
n=1157
Shared by G1,G2,G3
n=658
Unique G2
n=321
Intersection size
1428
1301
762
642
598
515
375
283
173
148
130
109
103
85
65
54
2
1
G3:down
G3:up
G2:down
G1:down
G2:up
G1:up
5,084
2,750
2,334
2,555
1,420
1,323
2,364
1,135
1,041
Set size(x10³)
M
G1
G2
G3
mean std gene expression
Cell lines grouped by sex and XCI status
B
group 3 log₂(fold change)
group 1 log₂(fold change)
larger effect
in group 3
in group 1
C
Differentiation efficiency for iPSC−derived endoderm
0.84
0.046
0.033
0.18
0.077
0.51
Male
Group1
Group2
Group3
D
Male−biased autosomal genes
logFC Male vs Female (group 1)
7.9e−08
yes
no
XIST-binding
E
differentially expressed genes
male - female (group 1)
male - female (group 2)
relative enrichment at hypermethylated sites
hypermethylation in progressive transitions of XCI erosion
expression
female-biased
male-biased
F
differentially expressed genes
male - female (group 1)
male - female (group 2)
male - female (group 3)
relative enrichment at hypomethylated sites
hypomethylation in progressive transitions of XCI erosion

Fig. S8

A

B

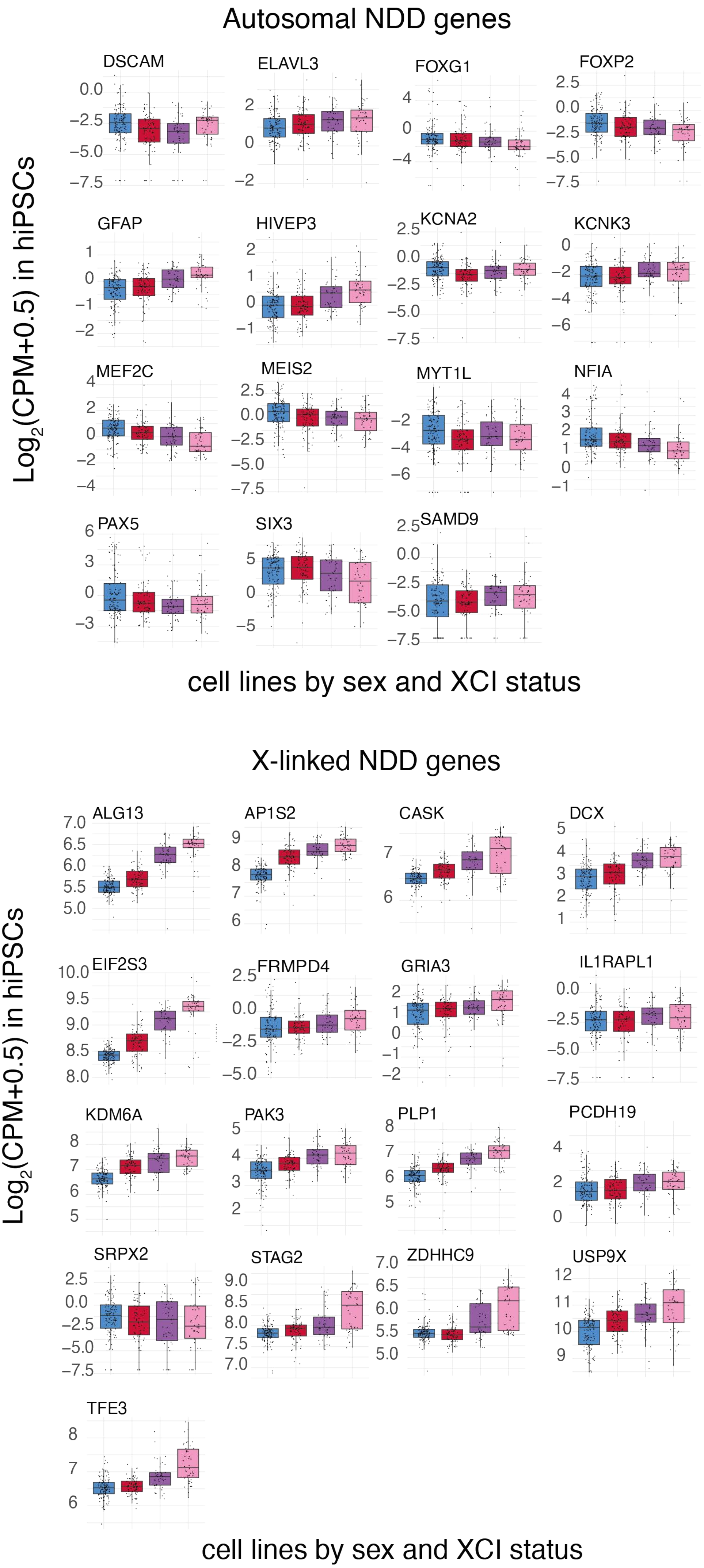
Autosomal NDD genes
DSCAM
ELAVL3
FOXG1
FOXP2
GFAP
HIVEP3
KCNA2
KCNK3
MEF2C
MEIS2
MYT1L
NFIA
PAX5
SIX3
SAMD9
Log_2(CPM+0.5) in hiPSCs
cell lines by sex and XCI status
X-linked NDD genes
ALG13
AP1S2
CASK
DCX
EIF2S3
FRMPD4
GRIA3
IL1RAPL1
KDM6A
PAK3
PLP1
PCDH19
SRPX2
STAG2
ZDHHC9
USP9X
TFE3
Log_2(CPM+0.5) in hiPSCs
cell lines by sex and XCI status

Fig. S9

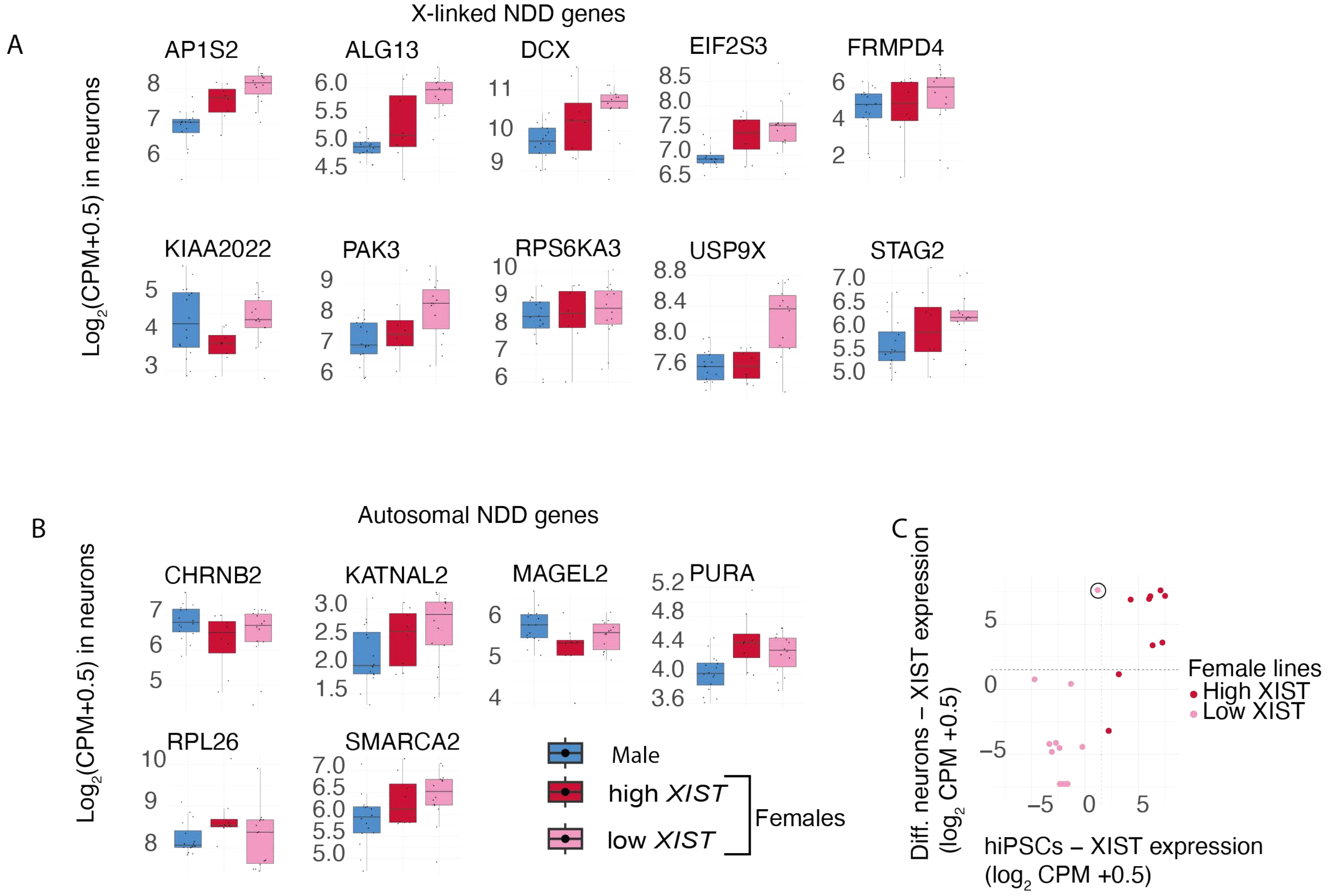
X-linked NDD genes
A
AP1S2
ALG13
DCX
EIF2S3
FRMPD4
KIAA2022
PAK3
RPS6KA3
USP9X
STAG2
Log2(CPM+0.5) in neurons
B
Autosomal NDD genes
CHRNB2
KATNAL2
MAGEL2
PURA
RPL26
SMARCA2
Log2(CPM+0.5) in neurons
Male
high XIST
low XIST
Females
C
Female lines
High XIST
Low XIST
Diff. neurons – XIST expression (log2 CPM +0.5)
hiPSCs – XIST expression (log2 CPM +0.5)
