# Supplementary material for: X-chromosome inactivation in human iPSCs provides insight into X-regulated gene expression in autosomes
Source: Genome Biol. 2024 May 31;25:144. doi: 10.1186/s13059-024-03286-8 (PMC11143737; doi:10.1186/s13059-024-03286-8)
Supplement: Supplementary file 3 — Additional file 3: Review_history. [file 13059_2024_3286_MOESM3_ESM.docx]

**1^st^ round**

**Reviewer 1**

-The term of "escapee" generally means escaped genes from Xi (i.e. biallelic expression status with XCI). It is different from de-repressed genes by erosion XCI. Erosion is accompanied by XIST loss, but escapee is not so. The authors should use the term correctly. The authors seem to be confused the differences of "erosion" and "escapee". Therefore, the current data set using "bulk" but not single cell levels cannot support their conclusion. It would be interesting how XIST expression levels correlates with ASE. However, it just might indicate the outcome of erosion, having been shown in previous studies (Vallot et al. Cell Stem Cell. 2015, Mekmohubad et al. Cell Stem Cell. 2012. Motosugi et al. Cell Rep. Methods. 2022).

-A major weak point in this study is lack of robust XCI definition. The clonality was evaluated by XIST ASE assay, this is nice approach to determine XCI allelic selection. However, If the authors want to claim that XIST expression level is an enough parameter to show the degree or status of "escapee", they must exclude possibility that "escapee" are not resulted from heterogeneity population of XIST loss (i.e. escapee is independent of XIST loss like erosion). The examination of escapee is essential at single cell resolution.

**Reviewer 2**

The work by Topa, et al. is a fascinating study of great importance. The manuscript is well-structured and was a pleasure to read!

The authors have used existing data from the HipSci project, using 165 cell lines deriving from females and 117 cell lines deriving from males, to assess XCI erosion and gene-wise escape from XCI. Further, they expand on this to assess the effects on autosomal gene expression and genes relevant to neurodevelopmental disorders. Through this endeavour, not only have the authors contributed to our understanding of XCI erosion in hiPSC cells, but also how escape from XCI - an understudied area - impacts the rest of the genome.

They expand on what is already known about the erosion of XCI in hiPSCs and demonstrate XCI erosion happens progressively in cultures and cannot be detected by low XIST expression alone. Instead, using XIST expression together with ASE data to define genes escaping ASE, they describe an intermediate group of cells, which, though they still have considerable XIST expression, also have elevated numbers of genes escaping XCI. Indeed, the intermediate group has a similar number of genes escaping XCI as those cells with low XIST expression (typically defined as eroded XCI), though the level of escape is lower. Using the 3 groups of cells defined by XIST expression and escape from XCI, the authors next show how increased erosion of XCI (with and without reduced XIST expression, eg. Groups 2 and 3) impacts the rest of the genome and leads to greater sex-differences in autosomal gene expression. They therefore rightly highlight the importance of taking a genome-wide approach, including the X chromosome to assess escape from XCI, when studying sex-differences in hiPSCs. However, they also make the important observation that, when modelled correctly, the increased escape from XCI, which augments chromosomal differences between XY and XX cells, could in fact offer enhanced mechanistic insights into sex effects. This has implications for disease modelling in hiPSCs and gene expression analyses in somatic cells. Their conclusions are well-supported by their robust analyses, which include the initial assessment of the assumption of clonality in hiPSCs. As such, the study has great relevance to a broad audience of biologists and will improve the use of cellular models.

The methods are appropriate, though I believe an additional methods section describing the statistical analyses is required. I have two further queries regarding the methods:

1) The authors state in the methods that the RNA-Seq data was not aligned with a SNP-aware method, which could lead to reference-allele bias. Indeed, WASP-correction during alignment is thought to be best practice for ASE to avoid alignment bias at biallelic SNPs. Have they assessed any reference biases in the ASE data?

2) How did the authors reach the conclusion that escape should be defined by the ASE >0.1 threshold. Were other thresholds considered?

And a final minor comment:

3) page 20-21, "Genes underlying X-linked developmental disorders vary in escape" results section. There are some references that are in a different format (superscript numbers) compared to the rest of the manuscript (numbers in brackets). As these are not in the correct order, I believe the references are wrong, rather than this being a formatting error.

**Reviewer 3**

Overview
In the present manuscript, the authors profile the X-chromosome inactivation (XCI) status of 165 female human induced pluripotent stem cells (hiPSCs) of the HipSci project. Female hiPSCs frequently display erosion of XCI in culture, accompanied by a loss in Xist expression. This phenomenon hinders the effectiveness of hiPSCs for disease modeling, particularly in the context of X-linked disorders.
The manuscript presents a detailed view of the degree of escape of a large number of X-chromosomal genes across all female cell lines studied using allele-specific RNA-seq analysis. By exploring the relationship between Xist expression levels and escape from XCI, the authors are able to define three groups of female hiPSCs. One of these groups exhibits early signs of XCI erosion in the absence of Xist repression, highlighting the importance of investigating XCI status in female hiPCS, not just Xist expression levels. Another key finding of the paper is the fact that increased escape from XCI affected autosomal gene expression in a dose-dependent manner, thereby altering sex differences. Several observations described in the manuscript have already been reported previously (e.g. clustering of genes subject to erosion and erosion of the Xist-coated Xi; PMID: 25921272, 33910018). However, the large number of analysed cell lines will make it nevertheless a valuable resource to the fields of XCI and human stem cell research. Regrettably, the potential of the extensive dataset has not been fully exploited to generate new biological insights. In addition, a series of clarifications regarding some of the analyses are needed, as detailed below. We also propose additional analyses to further clarify and add significance to some of the findings.

Code Availability
The authors do not appear to have deposited original code on GitHub. This should be rectified as the availability of code is vital to the reproducibility of the study

Major Comments
1. The authors assess the clonality of the female cell lines by investigating the allelic expression of Xist. In clonal cell lines the allele-specific expression (ASE) of Xist should be (very close to) 0, indicating monoallelic expression. 20 cell lines showed an ASE>0.05 for Xist, indicating slight biallelic expression. The authors argue that this low level of biallelic expression has no effect on the allelic expression of X-linked genes, since differences in median ASE and the fraction of genes with ASE>0.1 were not significantly different between Xist ASE<0.05 and Xist ASE>0.05 (Figure 1e, S2d). However, in Figure 1e, a trend towards higher median ASE in cell lines with Xist ASE>0.05 is visible. We therefore suggest to either exclude these cell lines from the analyses or to provide further evidence that the low levels of biallelic Xist expression do not impact the results presented in the manuscript.
2. The authors perform a differential expression analysis between the three groups of female cell lines and male cell lines, to investigate how escape from XCI affects male-female differences. Interestingly, escape from XCI was found to affect expression of autosomes in a dose-dependent manner (Figure 5). For X-linked genes, the authors show that a higher degree of escape from XCI resulted in a more pronounced female-biased expression and less-pronounced levels of male-biased expression of X-linked genes (Figure 4). However, these observations are not investigated further. A functional analysis of the autosomal genes would be useful. To add further significance to these findings, we suggest performing a gene ontology (GO) enrichment analysis on genes differentially regulated by increased escape, to gain a better understanding of the actual biological processes which might be affected.
3. Previous work in mice (PMID: 30639215) has shown that X dosage has effects on the developmental state and differentiation efficiency of iPSCs. Could the authors investigate which developmental stages these three groups of cells with respect to XCI erosion represent? Analysis of marker genes or gene signatures might provide insights into this question. Moreover, comparison of their expression profile to published data collected from human embryos could be helpful.
4. Previous work in mouse and human cells has shown a link between X-dosage and global DNA methylation in pluripotent cells (PMID: 34107261, 16244654). Could this be the mechanistic explanation for the different expression profiles in the three groups?
5. A previous study (PMID: 25921272) in hESC has shown that genes in specific regions of the X chromosome are prone to erosion and that these regions are enriched for H3K27me3. Are the eroded regions described in the manuscript the same? Can the observed effects of erosion on autosomal gene expression be linked to specific genes that escape? In mice, a recent study has identified X-chromosomal genes that mediated X-dosage effect on autosomal gene expression (PMID: 33863351). Could these genes account for the observed effects?
6. In Figures 4b,c and 5b,c, absolute log2 fold changes between gene expression of female and male cell lines are shown. Taking the absolute value of the fold change limits interpretability of the analysis. Instead the actual values (not absolute) should be shown, as to not hide effects on differential up- or downregulation, which should facilitate the interpretation of the data.
7. We suggest performing a principal component analysis (PCA) on the female cell lines. It would be interesting to see if the three groups of cell lines defined in the manuscript would also cluster together using this different analysis approach.
8. Many figure legends need to be re-written as they lack adequate details in their current form, making it challenging to comprehend the data shown. This is particularly noticeable for Figure 1, but further figures need to be checked as well. Also clarity of axis labels could be improved (e.g. when indicating a fold change it must be clarified which parameters were compared).

Minor Comments
1. Figure 2a shows the fraction of genes with ASE>0.1 for Xist-high and Xist-low female lines. However, it is unclear how many genes were assessed in each cell line. This information should be provided, either as an additional layer in the current figure or with an additional plot in the supplements.
2. In the legend for Figure 2a, it says that a Wilcoxon test was performed. But within the figure it says "t-test" next to the p value.
3. In Fig. 1C it is unclear how "ASE per gene per cell line" was calculated, this should be documented in the figure legend or the methods section.
4. In Fig. 2B the Xist gene should be highlighted.
5. In Figure 3d, the percentage of genes that were inactive, escaped or showed variable escape were compared between female human tissues and the female hiPSCs. In its current form the figure is confusing and difficult to understand. It is not clearly described what the colors for "Escape", "Variable" and "Inactive" in the legend and the position of the same categories refer to. Since this is an important figure, this should be improved.
6. A figure title is missing for Figure 5.
7. The analysis in Figure 5B is confusing.
8. In line 117 it is stated that naive iPSC would lose Xist expression. This is not correct in humans, Xist is still expressed but does not completely silence the chromosome. The whole paragraph that follows is confusing.
9. In line 155 it is described how many genes were analysed across the cell lines, it would be useful to know how many genes could be analysed in an individual line.
10. Overall, the text could be condensed, since it is very technical at times and partially redundant.
11. The term "normative escape" is unclear.
12. A quantitative relationship between Xist levels and degree of escape was also found in a previous study (ref. 3) and should be referenced when discussing these results in line 256.
13. Regarding Fig. 6c it is stated on line 458 that in neurons the genes "displayed similar patterns of expression with loss of XiST as observed in hiPSCs." This does not become evident from the figure. What aspects are similar? Moreover, it would be useful to show Xist expression in the neuron samples.

**Authors’ response**

Reviewer #1:

-The term of "escapee" generally means escaped genes from Xi (i.e. biallelic expression status  with XCI). It is different from de-repressed genes by erosion XCI. Erosion is accompanied by  XIST loss, but escapee is not so. The authors should use the term correctly. The authors seem  to be confused the differences of "erosion" and "escapee". Therefore, the current data set using  "bulk" but not single cell levels cannot support their conclusion. It would be interesting how  XIST expression levels correlates with ASE. However, it just might indicate the outcome of

erosion, having been shown in previous studies (Vallot et al. Cell Stem Cell. 2015,  Mekmohubad et al. Cell Stem Cell. 2012. Motosugi et al. Cell Rep. Methods. 2022).

**Response:**

We thank the reviewer for raising this point. We agree that the used terminology was ambiguous  and did not distinguish between “escape” and erogenous “de-repression”. We have now  changed the terminology throughout the manuscript to reflect this difference.

We respectfully disagree with the reviewer on the ability to use bulk RNA sequencing (RNA-seq)  to study escape and de-repression in hiPSCs through measuring allele specific expression  (ASE). Unlike normally in human tissues that have random XCI, hiPSC culture have extremely  skewed XCI with > 90% of cells carrying the same inactive copy of X (1, 2), which is also  supported by the data in the manuscript (**Figure 1D** in the manuscript). The extreme skew  enables the use of bulk RNA-seq for determining the degree of bi-allelic expression in the X  chromosome. Further, this approach allows for a more complete coverage of the X chromosomal genes (on average 59 genes assessed per individual) compared to single cell  RNA-seq where the use of ASE for the detection of true bi-allelic expression in the X  chromosome at the level of an individual cell is more challenging due to the generally lower  sequencing coverage and the excess of monoallelic expression resulting from both diverse  biological and technical factors (1, 2).

To address the reviewer’s point, we have carefully studied the allelic expression of *XIST*,  transcribed solely from the inactive copy of X, to confirm that the cultures have a low level of  chimerism (Page 6, **Figure 1D,E** in the manuscript). We show that the potential low level  chimerism is not associated with median ASE or fraction of genes with ASE >10% in X  chromosome, supporting that the results are not driven by heterogeneous cell populations. We  have now added more clarity to the text to address this (Page 6). We also show that the ASE of X  chromosome genes follows a completely different distribution than observed for autosomes  (**Figure 1B vs 1C** in the manuscript). In mixed chimeric populations, the X chromosome ASE  would resemble that of autosomes. The bulk RNA-seq have several advantages over single cell  RNA-seq in clonal cell populations: It provides substantially higher sequencing coverage for  determining ASE per site, it provides a wider spread of sequence reads across transcripts, and  it has lower drop-out of detection than in single cell RNA-seq thus providing more  comprehensive view of ASE. For these reasons, we do not expect that the study conclusions  suffer from substantial biases caused by the bulk RNA-seq.

Finally, we agree with the reviewer that the level of ASE correlates with *XIST* level. We pointed  this out in the manuscript (**Figure 2A** in the manuscript). We agree that the increase in ASE is  due to erosion-linked de-repression. We have clarified this in the text by distinguishing *escape* and *de-repression* as per requested by the reviewer. We have included the suggested articles

as references (3-5). We have also included comparison results from differential expression and  methylation, *XIST* binding, and histone modifications to provide insight into the genomic  mechanisms driving the gene expression during XCI erosion in the studied lines.

-A major weak point in this study is lack of robust XCI definition. The clonality was evaluated by  XIST ASE assay, this is nice approach to determine XCI allelic selection. However, If the authors  want to claim that XIST expression level is an enough parameter to show the degree or status of  "escapee", they must exclude possibility that "escapee" are not resulted from heterogeneity

population of XIST loss (i.e. escapee is independent of XIST loss like erosion). The examination  of escapee is essential at single cell resolution.

**Response:**

We thank the reviewer for the opportunity to clarify. We used two well-established metrics to  define the degree of XCI in the female cell cultures: *XIST* mRNA level and the fraction of X  chromosome genes that show bi-allelic expression, both of which have been linked with XCI  erosion in cell cultures (3-8). This enabled us to distinguish cultures based on quantitative  differences in XCI. Importantly, we did not use *XIST* level alone to define the XCI status of the  lines. The used metrics are appropriate for estimating the level of XCI and de-repression in the  cultures. We agree that potential non-clonality of cultures may bias the ASE estimates, as we  have pointed out in the manuscript (Page 6, line 129), which may inflate the estimates of XCI  erosion in a culture. However, as pointed out in our previous response to bulk RNA-seq, we  have carefully compared the ASE of *XIST*, X chromosome genes, and autosomal genes (**Figures  1B,C,D,E** in the manuscript) to ensure that the results are not significantly biased by  heterogeneity of the cell population. We have now clarified this point in the text as well. We  found no evidence of significant bias caused by chimeric cell populations (**Figure 1E, Figure  S2C** in the manuscript). We used allelic expression of *XIST* to study whether all cells in the  culture express the same parental copy of *XIST* (**Figure 1D** in the manuscript). Because *XIST* is  expressed only from the inactive X chromosome, cultures where all cells have the same  inactive copy of X chromosome express the same allele of *XIST*, which is detected as  monoallelic expression of *XIST*. Cultures where a fraction of cells has the other inactive  parental copy of X, express two different alleles of *XIST*. The level of *XIST* ASE in these chimeric  cultures depends on the sizes of the two cell populations and their level of *XIST* expression. We  identified 20 lines that had *XIST* ASE >5%. This corresponds to a small fraction of cells with the  alternative inactive copy, as we describe in the manuscript (Page 6, line 136). However, the  fraction of cells with alternative inactive X was not enough to cause a substantial bias in the  ASE estimates. Given that *XIST* ASE can only be measured for lines with sufficient *XIST* expression, similar analysis is not possible for lines with low *XIST* expression. However, it is  reasonable and justified to assume that the lines with high and low levels of *XIST* have similar  fractions of chimeric cultures and therefore, the small chimeric populations would not lead to  major biases in the estimates between the female groups or invalidate the conclusions made in  the study. Importantly, because the *XIST* ASE is unavailable for the lines with low *XIST*,  excluding the lines with traces of bi-allelic expression of *XIST* from the lines with high  expression of XIST would result in a bias in the analysis. We agree with the reviewer that  defining the XCI status for each cell would require single-cell measurement. However, this is  not feasible nor necessary for the conclusions made in the study; the analyses are based on  robust statistical estimates derived from cell populations. We have now added a more detailed  justification to the results section (Page 6, line 140). We have addressed this in the limitations  section of the manuscript (Page 23, line 594).

Reviewer #2:

The work by Topa, et al. is a fascinating study of great importance. The manuscript is well structured and was a pleasure to read!

The authors have used existing data from the HipSci project, using 165 cell lines deriving from  females and 117 cell lines deriving from males, to assess XCI erosion and gene-wise escape  from XCI. Further, they expand on this to assess the effects on autosomal gene expression and  genes relevant to neurodevelopmental disorders. Through this endeavour, not only have the

authors contributed to our understanding of XCI erosion in hiPSC cells, but also how escape  from XCI - an understudied area - impacts the rest of the genome.

They expand on what is already known about the erosion of XCI in hiPSCs and demonstrate XCI  erosion happens progressively in cultures and cannot be detected by low XIST expression  alone. Instead, using XIST expression together with ASE data to define genes escaping ASE, they  describe an intermediate group of cells, which, though they still have considerable XIST  expression, also have elevated numbers of genes escaping XCI. Indeed, the intermediate group  has a similar number of genes escaping XCI as those cells with low XIST expression (typically  defined as eroded XCI), though the level of escape is lower. Using the 3 groups of cells defined  by XIST expression and escape from XCI, the authors next show how increased erosion of XCI  (with and without reduced XIST expression, eg. Groups 2 and 3) impacts the rest of the genome  and leads to greater sex-differences in autosomal gene expression. They therefore rightly  highlight the importance of taking a genome-wide approach,including the X chromosome to  assess escape from XCI, when studying sex-differences in hiPSCs. However, they also make  the important observation that, when modelled correctly, the increased escape from XCI,  which augments chromosomal differences between XY and XX cells, could in fact offer  enhanced mechanistic insights into sex effects. This has implications for disease modelling in  hiPSCs and gene expression analyses in somatic cells. Their conclusions are well-supported by  their robust analyses, which include the initial assessment of the assumption of clonality in  hiPSCs. As such, the study has great relevance to a broad audience of biologists and will  improve the use of cellular models.

**Response**

We thank the reviewer for their interest and encouraging comments.

The methods are appropriate, though I believe an additional methods section describing the  statistical analyses is required. I have two further queries regarding the methods:

**Response:**

We thank the reviewer for this suggestion. We have added a separate statistics section to  methods, where we describe the statistical tests used in the study (Page 24).

1) The authors state in the methods that the RNA-Seq data was not aligned with a SNP-aware  method, which could lead to reference-allele bias. Indeed, WASP-correction during alignment  is thought to be best practice for ASE to avoid alignment bias at biallelic SNPs. Have they  assessed any reference biases in the ASE data?

**Response**

We thank the reviewer for pointing this out. We agree that the chosen method for ASE may lead  to a reference allele bias. To address this and to study the potential reference allele bias we  used the WASP-correction, suggested by the reviewer, for three cell lines (one from each  female group) and compared the ASE estimates used in the manuscript to those obtained by  the WASP correction (**Figure 1 below**). Reassuringly the two methods returned a close to  identical ASE estimates (r ~1) for each female line, implying minimal effect from reference allele  bias at the level of an individual sample.

For extra assurance, we studied the reference allele bias by comparing the proportion of reads  that mapped to the reference allele from all reads across all female samples. A large bias to the  reference allele would lead to underestimating the degree of bi-allelic expression (i.e. smaller  ASE values), because in our approach the more lowly expressed allele is assigned to the Xi, i.e,  the numerator in the calculation of ASE. We found that on average at around 50% of the biallelic  sites in the X chromosome the reference allele was assigned as the Xi, as is expected when  there is no reference allele bias (see **Figure 2 below**). We, however, observed a significantly  higher proportion of variants with the reference allele assigned as the Xa in Group 3 compared  to Group 1 and Group 2 females, in line with a contribution from reference allele bias. This  stronger bias in Group 3 suggests that the true ASE is slightly higher in this group than the  estimated ASE. The bias was observed primarily for genes with a clear bi-allelic expression with  ASE ranging 0.3–0.5 (**Figure 2 below**). This suggests that in cases where a gene is highly  expressed from the Xi, our initial assumption that the lower expressed allele belongs to Xi may,  indeed, be wrong. Together these findings, nevertheless, indicate that the reference allele bias  does not markedly alter the fraction of genes identified to escape or become de-repressed (see  boxes in **Figure 1 above**) and therefore does not impact the main findings or conclusion from  the analysis. We have now added a note of the reference allele bias to the manuscript methods  section (Page 28, line 720) and supplemental figure (**Figure S5B** in the manuscript).

**Response:**

We thank the reviewer for the opportunity to expand on the chosen ASE>0.1 threshold. In short,  the ASE > 0.1 threshold was selected based on previous literature to facilitate comparison,  particularly with human tissues. We concur that the selection of ASE threshold is a critical step  in the study since it may influence the level of detected bi-allelic expression and the grouping of  the female lines based on the fraction of escaped genes. Reassuringly, we found that different  ASE thresholds returned highly similar results. To study the influence of the selected ASE  threshold on the results and study conclusions, we compared the fraction of genes identified  with biallelic expression using five increasing thresholds for ASE (ASE>0.05, ASE>0.1,  ASE>0.15, ASE>0.2, ASE>0.25) (**Figure 3 below**). We found that the fraction of bi-allelic genes  was correlated across the different thresholds for the cell lines, suggesting that the results are  robust across different ASE bins. Lowering the threshold to ASE>0.05 increased slightly the  fraction of genes identified with bi-allelic expression and higher thresholds reduced the fraction  of bi-allelically expressed genes, as expected. To study how the thresholds influenced the  grouping of the female lines, we repeated the k-means clustering with *XIST* mRNA level and the  five ASE thresholds. We found that all but the extreme ASE>0.05 and ASE>0.25 thresholds

produced an optimal number of 3 clusters with close similarity of sample assignments. Finally,  given that there were a few lines where we identified a small degree of bi-allelic expression of  *XIST* (ASE:0.05-0.1), we reasoned that setting the threshold to ASE>0.1, rather than, e.g.,  ASE>0.05, would serve to avoid bias from the small fraction of chimeric cell populations in the  studied cell lines. We have now added a more detailed description to the Materials and  Methods section about the selected ASE threshold (Page 28, line 712).

And a final minor comment:

3) page 20-21, "Genes underlying X-linked developmental disorders vary in escape" results  section. There are some references that are in a different format (superscript numbers)  compared to the rest of the manuscript (numbers in brackets). As these are not in the correct  order, I believe the references are wrong, rather than this being a formatting error.

**Response:**

We thank the reviewer for pointing out this important error in the formatting. We have now  checked the references and edited them in the correct format in the manuscript.

Reviewer #3

In the present manuscript, the authors profile the X-chromosome inactivation (XCI) status of  165 female human induced pluripotent stem cells (hiPSCs) of the HipSci project. Female  hiPSCs frequently display erosion of XCI in culture, accompanied by a loss in Xist expression.  This phenomenon hinders the effectiveness of hiPSCs for disease modeling, particularly in the  context of X-linked disorders.

The manuscript presents a detailed view of the degree of escape of a large number of X chromosomal genes across all female cell lines studied using allele-specific RNA-seq analysis.  By exploring the relationship between Xist expression levels and escape from XCI, the authors  are able to define three groups of female hiPSCs. One of these groups exhibits early signs of XCI  erosion in the absence of Xist repression, highlighting the importance of investigating XCI status

in female hiPCS, not just Xist expression levels. Another key finding of the paper is the fact that  increased escape from XCI affected autosomal gene expression in a dose-dependent manner,  thereby altering sex differences. Several observations described in the manuscript have already  been reported previously (e.g. clustering of genes subject to erosion and erosion of the Xist coated Xi; PMID: 25921272, 33910018). However, the large number of analysed cell lines will  make it nevertheless a valuable resource to

the fields of XCI and human stem cell research. Regrettably, the potential of the extensive  dataset has not been fully exploited to generate new biological insights. In addition, a series of  clarifications regarding some of the analyses are needed, as detailed below. We also propose  additional analyses to further clarify and add significance to some of the findings.

**Response:**

We appreciate the reviewer’s constructive comments. We have now added a substantial body  of new analyses in the manuscript that we address below in itemized responses to the  reviewer’s points. In short, we have included analyses of overlap of the differential expression  results and methylation, histone modifications, and XIST binding to illuminate genomic  mechanisms driving the identified gene expression differences. We believe this analysis broadly  addresses the reviewer’s comments of significance and uses the data set to its full potential.  We have also included a more thorough review of earlier literature including the articles  referred to by the reviewer.

Code Availability

The authors do not appear to have deposited original code on GitHub. This should be rectified  as the availability of code is vital to the reproducibility of the study

**Response:**

We thank the reviewer for pointing this out and apologize for the lack of transparency here. This  has now been fixed and all code used for the analysis can be found on GitHub at  https://github.com/handetopa/hipsci-xci

Major Comments

1. The authors assess the clonality of the female cell lines by investigating the allelic  expression of Xist. In clonal cell lines the allele-specific expression (ASE) of Xist should be (very  close to) 0, indicating monoallelic expression. 20 cell lines showed an ASE>0.05 for Xist,  indicating slight biallelic expression. The authors argue that this low level of biallelic expression  has no effect on the allelic expression of X-linked genes, since differences in median ASE and  the fraction of genes with ASE>0.1 were not significantly different between Xist ASE<0.05 and  Xist ASE>0.05 (Figure 1e, S2d). However, in Figure 1e, a trend towards higher median ASE in cell  lines with Xist ASE>0.05 is visible. We therefore suggest to either exclude these cell lines from  the analyses or to provide further evidence that the low levels of biallelic Xist expression do not  impact the results presented in the manuscript.

**Response:**

We thank the reviewer for their comment and for carefully inspecting the data. We agree that  there is a trend toward higher ASE of X-linked genes in the lines that show some bi-allelic  expression of *XIST* (ASE>0.05). This may result in biasing the ASE estimates for the X-linked  genes due to a chimeric cell population, although, reassuringly, we detected no evidence for  differences in the fractions of bi-allelic X-chromosomal genes between these two XIST ASE  groups (**Figure S2C** in the manuscript). We chose to not exclude these lines because we can  only detect bi-allelic expression of *XIST* for lines that have sufficient sequence coverage for ASE  analysis (i.e. sufficiently high expression of XIST, n=80); similar analysis is not possible for lines

with low XIST expression (N=85). E.g. none of the lines assigned to group 3 have XIST ASE  information. It is likely, however, that the low and high *XIST* lines have a similar proportion of  chimeric cultures. Therefore, the removal of the known chimeric cultures only from the female  group with high expression of *XIST* will certainly bias comparisons with low *XIST* female lines.  Further, we reason that chimerism in the high *XIST* female lines will lead to higher ASE  estimates in the high *XIST* group, which may in turn dilute the observed differences with low  *XIST* lines. Therefore, the potential bias is unlikely to drive the findings or conclusions in the  manuscript. We have now expanded the justification in the manuscript (Page 6, line 139).

2. The authors perform a differential expression analysis between the three groups of female  cell lines and male cell lines, to investigate how escape from XCI affects male-female  differences. Interestingly, escape from XCI was found to affect expression of autosomes in a  dose-dependent manner (Figure 5). For X-linked genes, the authors show that a higher degree of  escape from XCI resulted in a more pronounced female-biased expression and less pronounced levels of male-biased expression of X-linked genes (Figure 4). However, these  observations are not investigated further. A functional analysis of the autosomal genes would  be useful. To add further significance to these findings, we suggest performing a gene ontology  (GO) enrichment analysis on genes differentially regulated by increased escape, to gain a better  understanding of the actual biological processes which might be affected.

**Response**

We thank the reviewer for this insightful comment and have now performed analysis for  enrichment for gene ontologies to understand which biological processes are affected in the  iPSCs by the erosion of XCI. In this respect, we find the genes that become sex-biased only in  the comparisons between group 3 females and males (n=2729) the most interesting as these  are expected to reflect the biological processes affected by erosion rather than more normative  sex differences in hiPSCs. The enrichment analyses for these genes, however, revealed few  significant enrichments (three GO terms for downregulated, none for upregulated genes). The  genes downregulated in both group 3 and 2 females, however, displayed enrichments for  diverse developmental pathways and, e.g., included functions related to chromosome  segregation (**Table S7**), suggesting several potential functions for the genes affected by erosion.

3. Previous work in mice (PMID: 30639215) has shown that X dosage has effects on the  developmental state and differentiation efficiency of iPSCs. Could the authors investigate  which developmental stages these three groups of cells with respect to XCI erosion represent?  Analysis of marker genes or gene signatures might provide insights into this question. Moreover,  comparison of their expression profile to published data collected from human embryos could  be helpful.

**Response:**

We thank the reviewer for pointing this out. We agree that a more careful assessment of the  association of the XCI states to differentiation efficiency is an important aspect. To address  this, we studied the endodermal differentiation efficiency recorded previously for the same  lines (9) with respect to the XCI erosion status. We found that differentiation efficiency was

slightly lower in group 2 and 3 lines with eroded XCI compared to group 1 females (**Figure 4  below**). However, the analysis revealed substantial line-to-line variability, suggesting  alternative mechanisms underlying the differentiation ability in addition to XCI status. We also  studied the expression of canonical stem cell marker genes (OCT4, SOX2, NANOG, **Figure 5**

**below)** between the different groups but found no statistical differences. Finally, as suggested  by the reviewer, we studied the expression levels of 32 pluripotency critical genes identified  from human embryonic stem cells (10) in the different female groups. We found no systematic  impact on these genes. Out of the 32 genes, 5 were downregulated and 7 were upregulated in  group 3 compared to group 1 female lines, suggesting that some pluripotency programs may be  altered by the erosion. Intriguingly, we also found that the X-linked *BCOR* was de-repressed in  the female hiPSCs. Previously heterozygous loss of *BCOR* has been linked with reduced  differentiation efficiency (9). It is tempting, therefore, to speculate that re-activation of this  same gene might have beneficial effects on differentiation as well. We have now included these  insights to the manuscript (Page 23, line 582).

4. Previous work in mouse and human cells has shown a link between X-dosage and global  DNA methylation in pluripotent cells (PMID: 34107261, 16244654). Could this be the  mechanistic explanation for the different expression profiles in the three groups?

**Response**

We thank the reviewer for this very insightful comment. We have now studied the overlap  between DNA methylation at gene promoters and de-repressed X-linked genes, as well as  differentially expressed genes in the autosomes, to explore whether the gene expression  differences are associated with hyper- and hypomethylation changes that take place at  different stages of XCI erosion (11). A comparison to methylation changes associated with five  consecutive transitions (1–5) in progressive stages of erosion of XCI in iPSCs (11) revealed a  significant enrichment for female-biased genes in X chromosome for early XCI erosion  associated hypomethylation (transition 1 out of 5, **Figure 6 below**). A trend for enrichment for  hypomethylated sites marking more advanced stages of XCI erosion (transitions 2 and 3) was  present in group 2 and group 3 with a higher degree of de-repression, in line with more  advanced XCI erosion. This suggested that lines were at a stage of XCI erosion consistent with  transitions 1 to 3 but had not reached transitions 4 and 5. This was further supported by  enrichment for transition 1 hypomethylated sites to genes that were found upregulated in group  2 and group 3 compared to group 1 and a trend for enrichment for upregulated genes in group 3  for hypomethylated sites related to transition 2 and 3.

In autosomes, no enrichment was found for hypomethylation with female-biased genes,  although the male-biased genes were significantly depleted from hypomethylated sites.  Similarly, we observed that hypermethylation of autosomes associated with XCI erosion, transitions 2 and 3 were enriched for male-biased genes in group 3 female lines. Together these

results suggest that male-female differences were in part driven by hypomethylation in X  chromosome and global methylation changes in autosomes that were consistent with partial  XCI erosion of the hiPSCs.

We have now added a paragraph describing these findings in the manuscript (Pages 16-17,  **Figure 5D, Figure S7D**)

5. A previous study (PMID: 25921272) in hESC has shown that genes in specific regions of the  X chromosome are prone to erosion and that these regions are enriched for H3K27me3. Are the  eroded regions described in the manuscript the same? Can the observed effects of erosion on  autosomal gene expression be linked to specific genes that escape? In mice, a recent study has  identified X-chromosomal genes that mediated X-dosage effect on autosomal gene expression  (PMID: 33863351). Could these genes account for the observed effects?

**Response**

We agree with the reviewer that studying the genomic mechanisms driving the gene expression  differences is important. Motivated by the suggestion, we have now also studied the  relationship of the X chromosome gene expression findings with the epigenetic landscape of  XCI using data for histone modifications for repressive heterochromatin marks H3K27 and  H3K9 trimethylation (H3K27me_3_, H3K9Me_3_) (3). We used ChIPseeker R package to annotate the  ChIP-seq histone peaks for H3K27me_3_ and H3K9Me_3_ at gene promoters (+/- 3kb from  transcription start site) and gene body (5’ UTR, 3’ UTR, exon, intron). We then used the  hypergeometric test to study the enrichment of sites with repressive H3K27me_3_ and  differentially expressed genes. Reactivated genes during XCI erosion were previously reported  to be enriched for sites with repressive H3K27me_3_ in normal inactivated cells (XaXi) removed  during erosion, while H3K9me_3_ is unaffected by XCI erosion (3). In line with the previous reports,  we observed that genes upregulated with advanced stages of XCI erosion in group 2 and group 3  female lines, were enriched for sites with repressive H3K27me_3_ histone marks in normal  inactivated cells (XaXi), but not for H3K27me_3_ histone marks in eroded lines (XaXe), suggesting  that removal of repressive histone marks were driving in part the observed gene expression  differences associated with XCI erosion (**Figure 7 below**). By contrast, no enrichment was  detected for H3K9me_3_. We have included these findings in a supplementary figure (**Figure S6  A,B**) and in the text (Pages 14-15).

Finally, we considered a hypothesis that the loss of *XIST* binding at autosomes in eroding  female lines was driving the attenuation of some male-biased expression in the lines. Recently,  *XIST* was shown to directly regulate the expression of autosomal genes *in trans* in naïve stem  cells through diffused binding to autosomes (12). Excitingly, we found that autosomal genes  with male-biased expression compared to group 1 female lines with high XIST level were  enriched for *XIST* binding. The enrichment disappeared for female lines with low level of *XIST*,  suggesting that reduced *XIST* levels in groups 2 and 3 lead to loss of XIST binding at autosomal  genes showing typically male-biased expression. A comparison of the male-female difference  for the 55 genes underlying the enrichment revealed a decreasing sex-difference by XIST  expression for the group 1, group 2 and group 3 females (**Figure 8 below**). Taken together, the  new results provide compelling evidence for multiple genomic mechanisms driving the XCI  erosion-associated gene expression differences and provide insight into X chromosome  regulated autosomal gene expression. We have now included these results in the manuscript  (Pages 17-18 and **Figure 5F,G**).

We thank the reviewer for pointing us to the interesting recent paper by Genolet et al (13). We  have now investigated how the human orthologs for these two mouse X-chromosomal genes,  Dusp9 and Klhl13, identified in that study show up in our data. DUSP9 displayed no evidence for  gene expression differences between the male and female lines or between the three groups of  female lines with different degrees of XCI erosion. However, KLHL13 was, indeed, female biased across all the comparisons and its expression appeared to increase with de-repression.  The ASE data further shows that the gene becomes de-repressed with XCI erosion (clear  biallelic expression in group 3 females) which may partly account for the increased female biased expression in the eroded lines. ASE data was not available for DUSP9. Given these  results, it is possible that KLHL13 is one of the contributors to the autosomal sex biases also in  human iPSCs, and that the de-repression of this gene further enhances these sex differences.  We have added this to the discussion section (Page 23) of the manuscript.

6. In Figures 4b,c and 5b,c, absolute log2 fold changes between gene expression of female  and male cell lines are shown. Taking the absolute value of the fold change limits  interpretability of the analysis. Instead the actual values (not absolute) should be shown, as to  not hide effects on differential up- or downregulation, which should facilitate the interpretation  of the data.

**Response**

We thank the reviewer for pointing this out. We agree that looking at absolute values will  remove the directionality of the effects that can complicate the interpretation. We had used  absolute values to emphasize the magnitude of the effect rather than the direction of the effect  and to reduce the visual complexity associated with multiple similar plots. We have now  changed the presentation to scatter plots that conserve the direction of the effects to show that  sex effects gained with XCI erosion and that are shared between group 2 and group 3 females  (n=2,018 genes in **Figure 5A** in the manuscript) have larger effect sizes in group 3 than in group  2 and the weakest effects in group 1 (**Figure 5B and C** and **Figure S7B** in the manuscript),  demonstrating a dose-dependent relationship with XCI erosion. Furthermore, we have added a  violin plot of sex effects that are lost in iPSCs with XCI erosion (**Figure 5E** in the manuscript) for  down and upregulated genes.

7. We suggest performing a principal component analysis (PCA) on the female cell lines. It

would be interesting to see if the three groups of cell lines defined in the manuscript would also  cluster together using this different analysis approach.

**Response**

We agree with the reviewer that reducing the dimensionality of the gene expression matrix with  PCA is an important and informative analysis. We performed a PCA separately for X  chromosome and autosome genes (**Figure 9 below**). For the X chromosome, the first  component captures variance in expression associated with sex (male/female) and three  groups of female lines that explains 14.5% of the total variance in X-linked gene expression.  This clustering aligns with the substantial changes that XCI erosion induces on the X  chromosome gene expression. For autosomes, the male and the three groups of female lines  are not associated with the two primary principal components (PC1 and PC2) that explain  20.5% of the total variance in autosomal gene expression, suggesting that although we see  clear autosomal effects related to the erosion of XCI there remains substantial variability due to  other factors in the autosomal gene expression. We have now added a PCA analysis to the  results section of the manuscript (Page 12, **Figure3C & Figure S5C** in the manuscript).

8. Many figure legends need to be re-written as they lack adequate details in their current  form, making it challenging to comprehend the data shown. This is particularly noticeable for  Figure 1, but further figures need to be checked as well. Also, clarity of axis labels could be  improved (e.g. when indicating a fold change it must be clarified which parameters were  compared).

**Response**

We appreciate the criticism and have reviewed and modified the figure legends with added  details as requested by the reviewer.

Minor Comments

1. Figure 2a shows the fraction of genes with ASE>0.1 for Xist-high and Xist-low female lines.  However, it is unclear how many genes were assessed in each cell line. This information should  be provided, either as an additional layer in the current figure or with an additional plot in the  supplements.

**Response**

We thank the reviewer for this suggestion. The number of informative genes (with at least 10 cell  lines with ASE data) in each cell line is shown as bar plots in **Figure S3**(ii). Additionally, we  provided this information in Supplementary Table S1. The median number of genes with ASE  data was 59 per line (range 2–79 genes). We have now added this to the text (Page 8, line 188),  and in the figure legend we refer to **Figure S3** for the number of informative genes.

2. In the legend for Figure 2a, it says that a Wilcoxon test was performed. But within the figure  it says "t-test" next to the p value.

**Response:**

We thank the reviewer for spotting this. We have now updated the figure with the p-value of the  Wilcoxon rank-sum test.

3. In Fig. 1C it is unclear how "ASE per gene per cell line" was calculated, this should be  documented in the figure legend or the methods section.

**Response:**

We thank the reviewer for pointing out that this may sound unclear. In Figure 1B and 1C, we  essentially plotted the distributions of ASE across all sites from all samples which had the ASE data  available in different groups (ASE was calculated for each biallelic site by dividing the number of  reads mapped to the minor alleles to the number of reads mapped to both alleles, as explained in  the ASE analysis methods section). To be clearer, we have now added the number of genes which  had ASE data available in each group in the figure legends.

4. In Fig. 2B the Xist gene should be highlighted.

**Response:**

We have now highlighted *XIST* in the figure.

5. In Figure 3d, the percentage of genes that were inactive, escaped or showed variable  escape were compared between female human tissues and the female hiPSCs. In its current  form the figure is confusing and difficult to understand. It is not clearly described what the  colors for "Escape", "Variable" and "Inactive" in the legend and the position of the same  categories refer to. Since this is an important figure, this should be improved.

**Response:**

We agree with the reviewer that the figure was not easy to understand. We have now clarified  the presentation by making a clearer distinction between the XCI status classifications from  human studies (escape, variable, inactive) and the pattern of bi-allelic expression in the three  groups of hiPSC female lines (the naming changed into bi-allelic, variably bi-allelic, and  monoallelic). These changes hopefully better convey 1) the overall increase in bi-allelic X chromosomal expression with XCI erosion consistently detected in all the cell lines, and 2) the  large overlap between established escape genes in human tissues and genes with bi-allelic  expression in hiPSC lines in a manner where only few known escapees are monoallelic in group  3 females. We have now also clarified this in the results section (Pages 12-13).

6. A figure title is missing for Figure 5.

**Response:**

We thank the reviewer for spotting this error. We have now added a title: “XCI erosion drives sex  differences in autosomal genes enriched for hypermethylation and XIST-binding regions.” to the  figure.

7. The analysis in Figure 5B is confusing.

**Response:**

We appreciate the reviewers' criticism and agree. We have now clarified the analysis and  changed the presentation for clarity. We now analyze the magnitudes of the effect sizes for  genes that are shared by group 2 and group 3 females (n=2018 genes) defined by the violet  vertical bar graphs in **Figure 5A** in the manuscript. We have changed the presentation to scatter  plots that preserve the direction of the effects in **Figure 5B and C** for the shared genes. The  figures show that the magnitudes of sex differences increase for these genes in group 3 with  increased XCI erosion. In **Figure 5 E,** we show genes that are unique to group 1 but have a  shared direction of effect in the eroded lines (n=672 genes) and have a dose-dependent  decrease in the eroded lines. The violin plot has been changed to show the directions of effects,  as per requested by the reviewer.

8. In line 117 it is stated that naive iPSC would lose Xist expression. This is not correct in  humans, Xist is still expressed but does not completely silence the chromosome. The whole  paragraph that follows is confusing.

**Response:**

We thank the reviewer for pointing this out. We agree that the section is not needed, and we  have now removed the paragraph from the text.

9. In line 155 it is described how many genes were analysed across the cell lines, it would be  useful to know how many genes could be analysed in an individual line.

**Response:**

We agree with the reviewer that it is important to understand the number of genes analyzed in  each line. We have now added a median and range of the number of genes analyzed for each  line (Pages 5 and 8). For all 411 X-linked genes we observed a median 76 genes with ASE data

per lines (Range: [2, 102]). For the 139 genes with at least 10 lines with ASE data the median  number of informative genes was 59 (Range: [2, 79]).

10. Overall, the text could be condensed, since it is very technical at times and partially  redundant.

**Response:**

We thank the reviewer for this suggestion. We have done substantial revision of text throughout  the results section to reduce repetition.

11. The term "normative escape" is unclear.

**Response:**

We agree. We have clarified this sentence by distinguishing the “de-repression” of genes that  once inactive are reactivated, from genes that “escape” i.e., remain expressed from both  copies during XCI. Consequently, we have modified the “normative escape” as “escape”  throughout the manuscript.

12. A quantitative relationship between Xist levels and degree of escape was also found in a  previous study (ref. 3) and should be referenced when discussing these results in line 256.

**Response:**

We thank the reviewer and agree. We have now added the reference appropriately.

13. Regarding Fig. 6c it is stated on line 458 that in neurons the genes "displayed similar  patterns of expression with loss of XiST as observed in hiPSCs." This does not become evident  from the figure. What aspects are similar? Moreover, it would be useful to show Xist expression  in the neuron samples.

**Response:**

We agree with the reviewer that language is not specific. What we have meant by “similar  pattern” is that the expression of these genes increases with loss of XIST in the lines, so that the  neuron lines with lowest XIST levels also have a higher expression of the disease genes as  iPSCs. We have now modified the text to be more specific. We have added neuronal XIST  expression to the supplementary table (**Figure S9C** in the manuscript).

**References**

1. Larsson AJM, Ziegenhain C, Hagemann-Jensen M, Reinius B, Jacob T, Dalessandri T, et  al. Transcriptional bursts explain autosomal random monoallelic expression and affect allelic  imbalance. PLoS Comput Biol. 2021;17(3):e1008772.

2. Tukiainen T, Villani AC, Yen A, Rivas MA, Marshall JL, Satija R, et al. Landscape of X  chromosome inactivation across human tissues. Nature. 2017;550(7675):244-8. 3. Vallot C, Ouimette JF, Makhlouf M, Feraud O, Pontis J, Come J, et al. Erosion of X  Chromosome Inactivation in Human Pluripotent Cells Initiates with XACT Coating and Depends  on a Specific Heterochromatin Landscape. Cell Stem Cell. 2015;16(5):533-46. 4. Mekhoubad S, Bock C, de Boer AS, Kiskinis E, Meissner A, Eggan K. Erosion of dosage  compensation impacts human iPSC disease modeling. Cell Stem Cell. 2012;10(5):595-609. 5. Motosugi N, Sugiyama A, Okada C, Otomo A, Umezawa A, Akutsu H, et al. De-erosion of  X chromosome dosage compensation by the editing of XIST regulatory regions restores the  differentiation potential in hPSCs. Cell Rep Methods. 2022;2(12):100352. 6. Brenes AJ, Yoshikawa H, Bensaddek D, Mirauta B, Seaton D, Hukelmann JL, et al.  Erosion of human X chromosome inactivation causes major remodeling of the iPSC proteome.  Cell Rep. 2021;35(4):109032.

7. Silva SS, Rowntree RK, Mekhoubad S, Lee JT. X-chromosome inactivation and  epigenetic fluidity in human embryonic stem cells. Proc Natl Acad Sci U S A.  2008;105(12):4820-5.

8. Tchieu J, Kuoy E, Chin MH, Trinh H, Patterson M, Sherman SP, et al. Female human  iPSCs retain an inactive X chromosome. Cell Stem Cell. 2010;7(3):329-42. 9. Puigdevall P, Jerber J, Danecek P, Castellano S, Kilpinen H. Somatic mutations alter the  differentiation outcomes of iPSC-derived neurons. Cell Genom. 2023;3(4):100280. 10. Ghosh A, Som A. RNA-Seq analysis reveals pluripotency-associated genes and their  interaction networks in human embryonic stem cells. Comput Biol Chem. 2020;85:107239. 11. Bansal P, Ahern DT, Kondaveeti Y, Qiu CW, Pinter SF. Contiguous erosion of the inactive  X in human pluripotency concludes with global DNA hypomethylation. Cell Rep.  2021;35(10):109215.

12. Dror I, Chitiashvili T, Tan SYX, Cano CT, Sahakyan A, Markaki Y, et al. XIST directly  regulates X-linked and autosomal genes in naive human pluripotent cells. Cell.  2024;187(1):110-29 e31.

13. Genolet O, Monaco AA, Dunkel I, Boettcher M, Schulz EG. Identification of X chromosomal genes that drive sex differences in embryonic stem cells through a hierarchical  CRISPR screening approach. Genome Biol. 2021;22(1):110.

**2^nd^ round**

**Reviewer 1**

In the revision, the authors added newly focused on sex differences based on XCI status. however, this study just showed X-linked gene expression status and major findings in this study were already reported by others (Vallot et al. 2015., Mekmohubad et al. 2012. Motosugi et al. 2022).
This paper largely analyzed only hPSCs and there is no data showing biological meaning. For instance, in the section of “Genes underlying X-linked developmental disorders vary in escape in hiPSCs”, they showed some up-regulated genes with bi-allelic expression compared to male lines. Even if these genes are up-regulated in female hPSCs, what is the meaning for NDD? These up-regulation might be no functional significance for neurodevelopment. Using the similar approach, large scale computational study could suggest that the differentiation potential depends on BCOR gene status (Puigdevall et al., 2023, Cell Genomics). Given that most parts of this study were already reported and no biological experiments, it would be difficult to find novel genome biological phenotypes in this study.

**Reviewer 2**

The authors have sufficiently addressed my earlier comments in the revised manuscript. In particular, their assessment of potential reference allele bias has shown that their estimates of ASE in Group 3 cell lines is slightly underestimated and this can now be seen in FigureS5B. These results do not impact the main findings of the study.

**Reviewer 3**

Overall, the authors have addressed most of my concerns and added some interesting new analyses to the manuscript. However, there are some concerns remaining as outlined below.

1. Reviewer 1 had commented on the fact that inter-cellular heterogeneity in erosion could lead to the detection of supposedly dose-dependent effects of Xist in bulk assays. I think this is a valid point and I would suggest to discuss it in the manuscript. Group 1 and group 2 show different Xist levels and different levels of de-repression. In principle there are two scenarios that could explain this: (A) Cell lines in group 2 contain a subpopulation of cells that have lost Xist expression, resulting in X-linked gene derepression in this subpopulation of cells or (B) all cells in the lines in group 2 still express Xist, but at a lower level, resulting in less pronounced derepression in all cells. In scenario A group 2 would be a mix of cells from group 1 and group 3, scenario 2 would support partial erosion, while Xist is still expressed (which is suggested in the manuscript, if I understand correctly). Since scenario A should result in a linear relationship between Xist and derepression, maybe the quantitative relationship can be used to distinguish the two scenarios. For this it would be useful to show a scatter plot of Xist expression and mean ASE in linear scale and analyze whether the relationship is linear or not.

2. The revised manuscript contains new analyses of histone modifications and DNA methylation at derepressed genes. The main text should explain the analyses in more detail and make it clear that the data used is from different studies, in particular for the histone modifications this does not become clear. Moreover, only a statistical analysis of the data is provided in the figures. It would be helpful to see more direct evidence of the suggested relationship. Specifically, it might help to show the levels of H3K27me3 and H3K9me3 at the promoters of genes upregulated in group, 1,2 and 3, respectively. Similarly, for DNA methylation it would be useful to see how many of the genes upregulated in group 1,2 and 3 are hypomethylated in step 1,2,3,4 and 5 respectively.

3. For the analysis of histone marks, it is not clear whether the ChiP-seq signal used arises from both X chromosomes or profiles the inactive X specifically.

4. The analysis of Xist binding to male-biased autosomal genes is really interesting. How strongly male biased are the 55 identified genes compared to the other male-biased genes?

Minor comments

5. Fig. 3E is more clear with the modifications. I would just suggest one more clarification for both figure and text. In the figure legend, the legend title is “human escape from XCI”. I would suggest to replace this with “escape in human tissues”, since referring to “human” alone is confusing, since the entire paper is focused in the human system.

6. On p. 10 it is stated that “some epigenetic marks underlying XCI may be subject to removal during reprogramming…”. How this follows from the fact that BCOR is often bi-allelic and affects differentiation capacity is not clear.

7. The font sizes used in the figures could be more consistent.

8. In Fig. 3D (and maybe other figures) the label “standardized gene expression” is used. Does that mean “normalized”? If so, I would suggest to use that term instead.

9. In Fig. 4B+C it would be helpful to add a title to make clear what genes are shown in the each plot.

**Authors’ response**

Reviewer #1:

In the revision, the authors added newly focused on sex differences based on XCI status. however, this study just showed X-linked gene expression status and major findings in this study were already reported by others (Vallot et al. 2015., Mekmohubad et al. 2012. Motosugi et al. 2022). 
This paper largely analyzed only hPSCs and there is no data showing biological meaning. For instance, in the section of “Genes underlying X-linked developmental disorders vary in escape in hiPSCs”, they showed some up-regulated genes with bi-allelic expression compared to male lines. Even if these genes are up-regulated in female hPSCs, what is the meaning for NDD? These up-regulation might be no functional significance for neurodevelopment. Using the similar approach, large scale computational study could suggest that the differentiation potential depends on BCOR gene status (Puigdevall et al., 2023, Cell Genomics). Given that most parts of this study were already reported and no biological experiments, it would be difficult to find novel genome biological phenotypes in this study.

We thank the Reviewer for the time and for peer-reviewing our work. We are glad to hear that we were able to adequately address the comments and concerns raised earlier by Reviewer 1. We are sorry to hear that Reviewer 1 does not agree with us on the novelty and importance of the reported findings.

Reviewer #2:

The authors have sufficiently addressed my earlier comments in the revised manuscript. In particular, their assessment of potential reference allele bias has shown that their estimates of ASE in Group 3 cell lines is slightly underestimated and this can now be seen in FigureS5B. These results do not impact the main findings of the study.

We thank Reviewer 2 for their time and commitment for peer-reviewing our work and for the previous constructive criticism. We are glad to hear that we have adequately addressed the reviewers’ concerns.

Reviewer #3:

Overall, the authors have addressed most of my concerns and added some interesting new analyses to the manuscript. However, there are some concerns remaining as outlined below.

 We thank the reviewer for their time and commitment for peer-reviewing our work, and the constructive and helpful comments and suggestions. Below, we provide itemised responses to the concerns raised by the Reviewer.

1. Reviewer 1 had commented on the fact that inter-cellular heterogeneity in erosion could lead to the detection of supposedly dose-dependent effects of Xist in bulk assays. I think this is a valid point and I would suggest to discuss it in the manuscript. Group 1 and group 2 show different Xist levels and different levels of de-repression. In principle there are two scenarios that could explain this: (A) Cell lines in group 2 contain a subpopulation of cells that have lost Xist expression, resulting in X-linked gene derepression in this subpopulation of cells or (B) all cells in the lines in group 2 still express Xist, but at a lower level, resulting in less pronounced derepression in all cells. In scenario A group 2 would be a mix of cells from group 1 and group 3, scenario 2 would support partial erosion, while Xist is still expressed (which is suggested in the manuscript, if I understand correctly). Since scenario A should result in a linear relationship between Xist andderepression, maybe the quantitative relationship can be used to distinguish the two scenarios. For this it would be useful to show a scatter plot of Xist expression and mean ASE in linear scale and analyze whether the relationship is linear or not.

We thank the reviewer for this comment. We explored more the potential scenarios how XCI erosion could progresses in the cultures, as suggested by the reviewer. We compared the *XIST* expression and the mean ASE of the female lines. This comparison yielded similar pattern with XIST expression as seen for the fraction of de-repressed genes (ASE>0.1, Figure 3A in the manuscript). Indeed, the mean ASE and the fraction of genes with ASE>0.1 were strongly correlated (r=0.96), yielding highly similar patterns with *XIST* expression (see Additional File 1: Fig. S5A and Results section page 11). A careful examination of the relationship of mean ASE and the *XIST* levels suggested that *XIST* had strong negative effect on mean ASE at levels higher than 4 logCPMs that corresponded to the transition from group 1 to group 2. The negative relationship between XIST expression and mean ASE was weaker for the lower XIST levels (XIST< 4 logCPM) that coincided with the transition from group 2 to group 3 in the female lines. (The smaller difference in the mean ASE between group 2 and 3 than between group 1 and 2 is also detectible in Figure 3B of the manuscript). Fitting a sigmoid curve on the data for XIST expression and mean ASE suggested a mid-point for the linear change (inflection point) at 6.6 logCPM of *XIST* expression (with a slope of -0.78) that plateaued at mean ASE of 0.2 (the asymptote). In addition, we observed no association with *XIST* levels and mean ASE within any of the female groups. Nevertheless, beyond the ASE of X chromosome, the group 3 has marked changes in autosomal sex differences in gene expression compared to group 2 in line with progressive changes between the groups. Together these results imply that there is no simple linear relationship with the de-repression and *XIST* levels in the data. We have now included these new results in supplemental figure (Fig. S5B and S5D) and in the text in the Results section (page 11), as well as discussion section (page 23).

2. The revised manuscript contains new analyses of histone modifications and DNA methylation at derepressed genes. The main text should explain the analyses in more detail and make it clear that the data used is from different studies, in particular for the histone modifications this does not become clear. Moreover, only a statistical analysis of the data is provided in the figures. It would be helpful to see more direct evidence of the suggested relationship. Specifically, it might help to show the levels of H3K27me3 and H3K9me3 at the promoters of genes upregulated in group, 1,2 and 3, respectively. Similarly, for DNA methylation it would be useful to see how many of the genes upregulated in group 1,2 and 3 are hypomethylated in step 1,2,3,4 and 5 respectively.

We thank the reviewer for the comment. We have now added more detailed description of the analysis to the main text (page 15-17), as well as added additional figures to better visualize the numbers of genes overlapping the hypo- and hypermethylation sites (fig.S6E-G).
 
3. For the analysis of histone marks, it is not clear whether the ChiP-seq signal used arises from both X chromosomes or profiles the inactive X specifically.

We thank the reviewer for this point and have now clarified this in the text. The ChIPseq data used was obtained from both chromosomes.
 
4. The analysis of Xist binding to male-biased autosomal genes is really interesting. How strongly male biased are the 55 identified genes compared to the other male-biased genes?

 We thank the reviewer for this interesting question. Indeed, the male-biased genes bound by *XIST* had on average larger magnitudes of difference in expression than the male biased genes not bound by XIST. We have now included this results in supplemental figure (Fig. S7D) and text (page 19).

Minor comments
 
5. Fig. 3E is more clear with the modifications. I would just suggest one more clarification for both figure and text. In the figure legend, the legend title is “human escape from XCI”. I would suggest to replace this with “escape in human tissues”, since referring to “human” alone is confusing, since the entire paper is focused in the human system.

We agree with the reviewer. We have made this change accordingly.
 
6. On p. 10 it is stated that “some epigenetic marks underlying XCI may be subject to removal during reprogramming…”. How this follows from the fact that BCOR is often bi-allelic and affects differentiation capacity is not clear.

 We appreciate the reviewer pointing this out to us. We agree that the sentences are unclear, and we have fixed this now. By saying that “[…],*the frequent bi-allelic expression of human inactive genes in high XIST lines could indicate stem cell specific escape of these genes rather than de-repression, suggesting that some epigenetic marks underlying XCI may be subject to removal during reprogramming, and they may have roles in the stem cell state and differentiation capacity of the hiPSCs*.”

7. The font sizes used in the figures could be more consistent.

We appreciate the reviewer’s comment and have edited font sizes in the figures.

8. In Fig. 3D (and maybe other figures) the label “standardized gene expression” is used. Does that mean “normalized”? If so, I would suggest to use that term instead.

We thank the reviewer for raising this point. The standardized gene expression is mean standardized expression i.e. a z score and it is a different from normalized gene expression. We have clarified this in figure 3D legend.
 
9. In Fig. 4B+C it would be helpful to add a title to make clear what genes are shown in the each plot.

We thank the reviewer and agree. We have added a clarifying text in the figure with numbers of genes top help matching with figure 4 A.
